# Phenotypic and Transcriptomic Response of the Grasshopper *Oedaleus asiaticus* (Orthoptera: Acrididae) to Toxic Rutin

**DOI:** 10.3389/fphys.2020.00052

**Published:** 2020-02-21

**Authors:** Xunbing Huang, Shenjin Lv, Zehua Zhang, Babar Hussain Chang

**Affiliations:** ^1^College of Agriculture and Forestry Science, Linyi University, Linyi, China; ^2^State Key Laboratory for Biology of Plant Diseases and Insect Pests, Institute of Plant Protection, Chinese Academy of Agricultural Sciences, Beijing, China; ^3^Department of Entomology, Sindh Agriculture University, Tando Jam, Pakistan

**Keywords:** grasshopper, rutin, phenotype, transcriptomics, biopesticides

## Abstract

Rutin, a widely distributed phytochemical flavonoid, can be used to control insect pests. In this study, we studied the growth performance of the grasshopper *Oedaleus asiaticus* Bey-Bienko given xenobiotic rutin using feeding experiments and transcriptomic analysis. *O. asiaticus* had reduced body size, lower survival rate, and reduced growth performance when fed with xenobiotic rutin. Rutin-fed nymphs had large variation in gene expression profiles, with a total of 308 genes significantly upregulated and 287 genes downregulated. The upregulated genes were significantly enriched in stress resistance-, immune-, and detoxification-related biological processes and the Kyoto Encyclopedia of Genes and Genomes (KEGG) pathways. Downregulated genes mainly involved cuticle biosynthesis and nutrition metabolism-related pathways. The quantitative real-time PCR (qRT-PCR) analysis of 15 candidate genes also produced results consistent with the transcriptome data. These results suggested that grasshoppers’ capacity for biosynthesis and nutrition metabolism decreased, and stress resistance and metabolized capacity to toxic substances were significantly induced when *O. asiaticus* was fed on xenobiotic rutin. Rutin, as a phytotoxin, had detrimental effects and induced changes in gene expression profiles for *O. asiaticus*. This study can provide a molecular basis and offer future opportunities for the development of rutin-related insecticides and their application to grasshopper control.

## Introduction

The use of synthetic pesticides undoubtedly has contributed to the development of agriculture, but it has also caused problems, including environmental toxicity and residual properties in water, soil, and crops (Senthil-Nathan, 2013; Jallow et al., 2017). In addition, the indiscriminate use of synthetic pesticides generally results in insect pest resistance and resurgence. The hazardous effects on the environment associated with pesticides can endanger the sustainability of ecosystems (Senthil-Nathan, 2013; Cevizci et al., 2015). Hence, searching for ecofriendly biopesticides that are target specific, rapidly degradable, and low in toxicity is essential. Secondary metabolites derived from plants, such as alkaloids, flavonoids, terpenoids, and sterols, are important resources for such biopesticides (De Oliveira et al., 2014; Isman, 2014; Campos et al., 2018). These chemical compounds normally rapidly degrade and, as such, lack persistence in the ecosystem (Theis and Lerdau, 2003; Senthil-Nathan, 2013; Michael, 2018; Monsreal-Ceballos et al., 2018). Research concerning the effects of these compounds on insect pests can offer future opportunities in the development of new bioinsecticides.

Generally, many plant-derived compounds can be used as toxicants, and they have been shown to have adverse effects to insect pests (Leicach and Chludil, 2014; Mouden et al., 2017; Michael, 2018). Diet stress from toxic exposure can significantly affect herbivorous insects. Some toxic compounds can cause mortality or reduced growth of pest insects through feeding deterrence or oviposition deterrence (Tangtrakulwanich and Reddy, 2014). For example, chemical azadirachtin can produce intestinal lesions, destroy digestive enzyme systems, and eventually lead to insect death (Ley, 2005; Senthil-Nathan, 2013). Nicotine, matrine, and quercetin-based biopesticides can have sublethal, lethal, or other deleterious effects on coleopteran and lepidopteran pest species (Senthil-Nathan, 2013; Poreddy et al., 2015).

Insect herbivores have evolved multiple strategies to overcome these plant chemical defenses, including contact and ingestion avoidance, sequestration, excretion, target-site mutation, and degradation of the toxin (Després et al., 2007; Poreddy et al., 2015; Birnbaum et al., 2017). The related molecular processes of detoxifying mechanisms, transporters, immunity, and peritrophic membranes allow insects to develop resistance to plant toxic substances (Roy et al., 2016; Birnbaum et al., 2017). For example, molecular adaptation has allowed more robustly active digestive, protective, and detoxifying-related enzymes in herbivorous insects; this includes the rapid synthesis of hydrolase, antioxidase, or cyP450s (Després et al., 2007; Senthil-Nathan, 2013). Such physiological responses to xenobiotic chemicals are vitally important for insect survival and reproduction (Roy et al., 2016).

Rutin, a widely distributed flavonoid glycoside, generally can help plants resist insect attack (Chen et al., 2017). The extracted flavonoid rutin from plants has deleterious effects on the pest insects *Lymantria dispar*, *Spodoptera litura*, *Pectinophora gossypiella*, *Heliothis virescens*, *Spodoptera eridania*, and *Helicoverpa zea* (Simmonds, 2003; Salunke et al., 2005; Mesbah et al., 2007; Taggar and Gill, 2016). Although insect phenotypic responses to toxic rutin are well documented, the underlying molecular basis of gene regulation are not well understood. *Oedaleus asiaticus* Bey-Bienko (Orthoptera: Acrididae), a dominant grasshopper pest of northern Asian grasslands, is distributed throughout northern China. Plagues of this grasshopper can severely damage grassland production and ecology and can aggravate grassland degradation (Cease et al., 2012; Zhang et al., 2013; Huang et al., 2016). Given the significant economic and ecological impact of this grasshopper, understanding the mechanism of grasshoppers’ response to xenobiotic rutin can potentially improve management of the grasshopper.

In this study, we sought to reveal how xenobiotic rutin influences gene expression and the resulting phenotype of grasshopper *O. asiaticus*. Specifically, the transcriptomics underlying the manifold changes for toxic rutin were examined to explore the altered gene regulation. Our ultimate goal is to use such knowledge about phytochemicals and rutin-induced gene regulation to control insect pests.

## Materials and Methods

### Grasshopper Collection

Third-instar nymphs of *O. asiaticus* were collected using sweep nets from Xilin Gol League (43.968°N, 115.821°E), Inner Mongolia in northeastern China. These collected nymphs were maintained temporarily in metal frame cages (50 × 50 × 50 cm). Cages were then placed in an illumination incubator for 1 day under an artificial light regime (14 h light–10 h dark) at 28 ± 1°C and a relative humidity of 70 ± 1%. We used fresh-cut *Stipa krylovii* Roshev (the optimal food grass) to feed *O. asiaticus* nymphs until they were transferred to the feeding trial (see below).

### Feeding Trial

We investigated *O. asiaticus* growth performance when the nymphs were reared using rutin-treated foods. Before the artificial rutin-feeding trial, a total of 400 third-instar female individuals were selected and starved for 12 h before the trial. Because the gender of the early instars is difficult to identify, third-instar female individuals were selected based on external morphology of the reproductive system. All selected nymphs were assigned to 20 plastic cages (30 × 20 × 10 cm), with each cage including 20 individuals. We used the grass *S. krylovii*, the most favored food of *O. asiaticus*, to feed the assigned nymphs. *S. krylovii* was collected freshly from the field and trimmed to 10 cm, then treated with rutin. In previous studies, we found that ∼0.01% rutin content in food plant had significant adverse effects to *O. asiaticus* (Huang et al., 2017; Li et al., 2019). To further explore the grasshopper phenotypic and transcriptomic response to toxic rutin, we prepared 0.01% rutin solution using sterile water. For each cage, 100 ml prepared rutin solution was applied evenly to 100 g of fresh *S. krylovii*, and then provided to the grasshoppers. *S. krylovii* treated with only sterile water was used as the control (no rutin treatment) and was provided to the allocated cage using the same methods as above. Each treatment was replicated 10 times. The treated fresh *S. krylovii* was replaced every 24 h for each cage. Grasshoppers were cultivated in cages under an artificial light regime (14 h light–10 h dark) at a temperature of 28°C and relative humidity of 70%. Before the artificial feeding trial, another 30 *O. asiaticus* third-instar females were euthanized by diethyl ether and dried at 90°C for 24 h, after which they were individually weighed and the mean (third-instar body mass, milligram) determined to serve as baseline data. Once grasshopper nymphs were assigned to each cage, we inspected grasshopper survival and removed dead individuals daily. This artificial feeding trial lasted for 7 days. For each treatment, a total of 30 female nymphs were collected (three females per cage) on the seventh day, and the nymphs were then used for RNA-seq. The remaining surviving individuals at the last day were also euthanized and dried using the same method as above to determine their dry body mass (milligram). The increase in body mass (milligram) was calculated by subtracting the basic third-instar body mass from the grasshopper body mass of the feeding trial. Survival rate (%) was calculated by the number of surviving individuals on last day/number of initial third-instar individuals (*n* = 20). Growth rate (mg/day) was calculated by the body mass increase (milligram)/development time (7 days).

### RNA Preparation, Library Construction, and Transcriptome Sequencing

We collected three samples from each of our two treatments at the seventh day of the feeding trial. Each sample consisted of 10 female nymphs (one chosen randomly from each of the 10 replicates). Hence, six samples (three biological replicates from each treatment) were analyzed. Each sample consisted of 10 female nymphs (combined). The collected samples were named by abbreviating the grasshopper name and treatment followed by the sample number; OA_ CK-1, OA_ CK-2, OA_ CK-3, OA_ Rutin-1, OA_ Rutin-2, and OA_ Rutin-3.

We used TRIzol reagent (Invitrogen, CA, United States) to extract total RNA from each sample according to the manufacturer’s instructions. The RNA sample quality was monitored on 1% agarose gels and checked by the NanoPhotometer spectrophotometer (IMPLEN, CA, United States), Qubit RNA Assay Kit in the Qubit 2.0 Fluorometer (Life Technologies, CA, United States), and RNA Nano 6000 Assay Kit of the Agilent Bioanalyzer 2100 system (Agilent Technologies, CA, United States). High-quality RNA (1.9 < OD260/280 < 2.1; RNA integrity number *RIN* > 8.0) from each sample was used for complementary DNA (cDNA) library construction. Then, sequencing libraries were generated using the NEBNext Ultra RNA Library Prep Kit for Illumina (NEB, United States) following the manufacturer’s recommendations. Finally, the libraries were sequenced on the Illumina HiSeqTM 4000 platform (Illumina Inc., San Diego, CA, United States).

### Data Processing and Differential Expression Analysis

Raw reads in fastq format were first processed through an in-house perl script. Adapters and reads containing poly-N or of low quality were removed. Q20, Q30, GC content, and sequence duplication level of the clean data were used for data filtering. After filtering the raw reads, *de novo* assembly of the transcriptome was carried out using Trinity (Grabherr et al., 2011). Then, generated unigenes were used for BLASTX searches and annotated based on the following databases: NCBI non-redundant nucleotide database (Nt), Eukaryotic Ortholog Groups (KOG), National Center for Biotechnology Information (NCBI) non-redundant protein database (Nr), the Swiss-Prot protein database (Swiss-Prot), and the Kyoto Encyclopedia of Genes and Genomes (KEGG). All searches were performed with an *E* value < 10^–5^. In addition, we used the Blast2GO program (Conesa et al., 2005) to obtain the Gene Ontology (GO) annotation of unigenes.

The sequenced reads for each sample were remapped to the assembled transcriptome using SOAPaligner/soap2 (Li et al., 2009). Gene expression values were quantified as fragments per kilobase per million mapped reads (FPKM) by RNA-Seq by Expectation Maximization (RSEM) (Li and Dewey, 2011). Differentially expressed genes (DEGs) were detected by DESeq2 R package (Anders and Huber, 2010). Count matrix was obtained using *featureCounts* function in the Rsubread package (Liao et al., 2013). Transcripts with a minimum 2-fold difference (| log_2_.Fold_change| > 1) in expression and adjusted *P* < 0.05 were considered differentially expressed between the control and rutin-treated group.

### Functional Analysis of DEGs

Differentially expressed genes were annotated to the GO database by the GOseq R packages based on Wallenius non-central hypergeometric distribution (Young et al., 2010) and mapped to pathways in the KEGG database using KOBAS software (Mao et al., 2005). GO terms significantly enriched for DEGs were identified by hypergeometric tests using transcriptome background. The resulting *P* values were adjusted using the Benjamini–Hochberg (BH) method. A corrected *P* < 0.05 was used as the threshold for statistical significance.

### Quantitative Real-Time PCR Validation

Fifteen candidate DEGs (Supplementary Table S1) involved in grasshopper cuticle biosynthesis, nutrition metabolites, stress resistance, or detoxifying enzymes were chosen for validation using quantitative real-time PCR (qRT-PCR). These candidate genes were *FOXO* (FoxO protein, Cluster-11327.75652), *HSP 90* (heat shock protein 90, Cluster-11327.59722), *CYP450 6K1* (cytochrome P450 6 K1, Cluster-11327.54882), *CYP450 9E1* (cytochrome P450 9E1, Cluster-11327.70406), UGT 2C1 (UDP-glucuronosyltransferase 2C1, Cluster-11327.22262), *UGT 2B1* (UDP-glucuronosyltransferase 2B1, Cluster-11327.24983), *LPH* (Lactase-phlorizin hydrolase, Cluster-11327.46783), *BG* (beta-glucosidase, Cluster-11327.3892), *SOD* (superoxide dismutase, Cluster-11327.55763), *POD* (Peroxidase, Cluster-11327.56874), *CarE* (carboxylesterase, Cluster-11327.66426), *VG* (vitellogenin, Cluster-11327.56459), *LCP* (Larval cuticle protein 2, Cluster-11327.87505), *CS* (Chitin synthase 1, Cluster-11327.80081), and *CP* (Cuticle protein 7, Cluster-11327.93652).

Gene-specific primers (Supplementary Table S2) for qRT-PCR of those 15 genes were designed using the software Primer 3^1^. We collected one nymph on the last day of the feeding trial randomly from each replicate cage of the two treatments (20 samples). Total RNA was extracted using the same method as described above. The cDNA was synthesized using avian myeloblastosis virus (AMV) reverse transcriptase (Invitrogen, Carlsbad, CA, United States) according to the manufacturer’s instructions. The same qRT-PCR procedure described by Li et al. (2019) were used to detect the gene expression. Relative gene expression levels were analyzed using the 2^–Δ^ ^Δ^ ^CT^ method, with β-actin as reference gene (Zhang et al., 2015; Huang et al., 2017; Li et al., 2019). Expression values were adjusted by setting the expression of controls to be 1 for each gene. All methods and data collections in qRT-PCR followed the Minimum Information for Publication of Quantitative Real-Time PCR Experiments (MIQE) guidelines. All qRT-PCRs for each gene of 20 samples (10 biological replicates for each treatment) used three technical replicates per experiment.

### Data Analysis

Student’s *t* test were used to compare grasshopper growth performance variables (survival rate and growth rate) in feeding trial, and the relative gene expression by qRT-PCR. SAS version 8.0 were used for all analyses.

## Results

### Growth Performance of *O. asiaticus* to Xenobiotic Rutin

**FIGURE 1:**
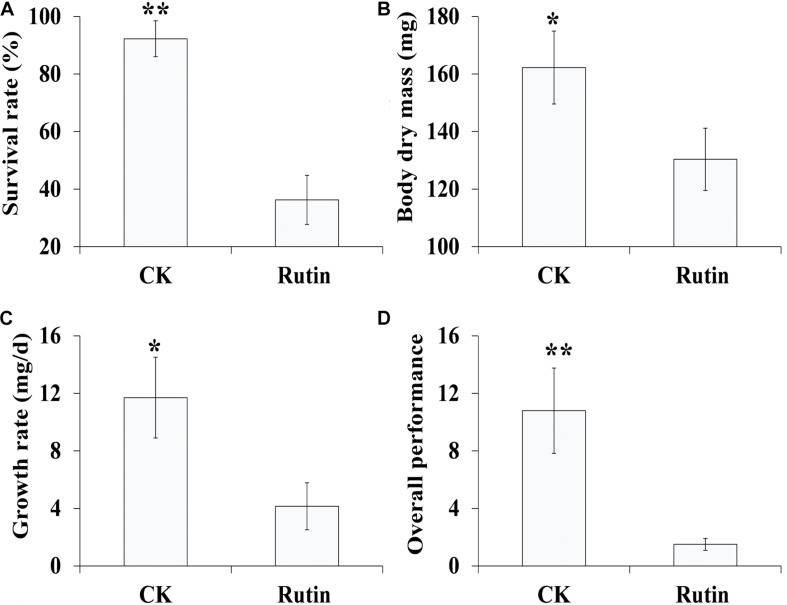
**(A)** *Oedaleus asiaticu*s mean% survival rate ± SD, **(B)** mean dry mass (mg ± SD), **(C)** mean growth rate (g/day ± SD), and **(D)** overall performance (± SD) when fed by rutin-treated foods. *P < 0.05, **P < 0.01 (Student’s *t* test).

In the artificial feeding trial, we examined *O. asiaticus*’ growth performance in response to xenobiotic rutin (Figure 1). *O. asiaticus* feeding on xenobiotic rutin had significantly reduced survival rate (Figure 1A), body mass (Figure 1B), growth rate (Figure 1C), and overall performance (Figure 1D) compared to CK. This indicates that feeding on rutin has detrimental effects for *O. asiaticus* growth and development.

### Transcriptome Analysis

To investigate the response of grasshoppers to xenobiotic rutin, the collected samples (three biological replicates) were analyzed by RNA-seq. Sequencing the transcriptomes of *O. asiaticus* fed on rutin generated 174,065,253 bases (Table 1). All six libraries were good quality, with Q20 and Q30 values >95 and 90%, respectively, with a reasonable GC content (Table 1 and Supplementary Table S3). The total numbers of unigenes was 133,144, with a mean length of 1,037 bp (Table 1).

**TABLE 1:** Statistics for the assembled sequences.

**Group name**	**Number**
Total assembled bases	174,065,253
Total number of unigenes	133,144
GC percentage (%)	46.68
Unigene N50 (bp)	1,705
Unigene N90 (bp)	533
Maximum unigene length (bp)	28,340
Minimum unigene length (bp)	2,250
Average unigene length (bp)	1,307

Of the 133,144 unigenes, a total of 67,101 (50.39%) were annotated in at least one database. Among these, 54,162 (40.67%) were successfully annotated by NCBI Nr, 17,040 (28.14%) by Swiss-Prot, 45,846 (34.43%) by GO, 19.392 (14.56%) by KEGG, 26,428 (19.84%) by KOG, and 17,040 (12.79%) by NCBI Nt (Supplementary Table S4). Transcriptome data have been submitted to the NCBI SRA database (accession number SRP072969). The majority of the sequences matched insect proteins, with the most abundant matching *Zootermopsis nevadensis* (30.6%), *Stegodyphus mimosarum* (4.8%), *Tribolium castaneum* (5.5%), *Acyrthosiphon pisum* (3.2%), and *Lasius niger* (2.8%) (Supplementary Figure S1).

### Differentially Expressed Genes

**FIGURE 2:**
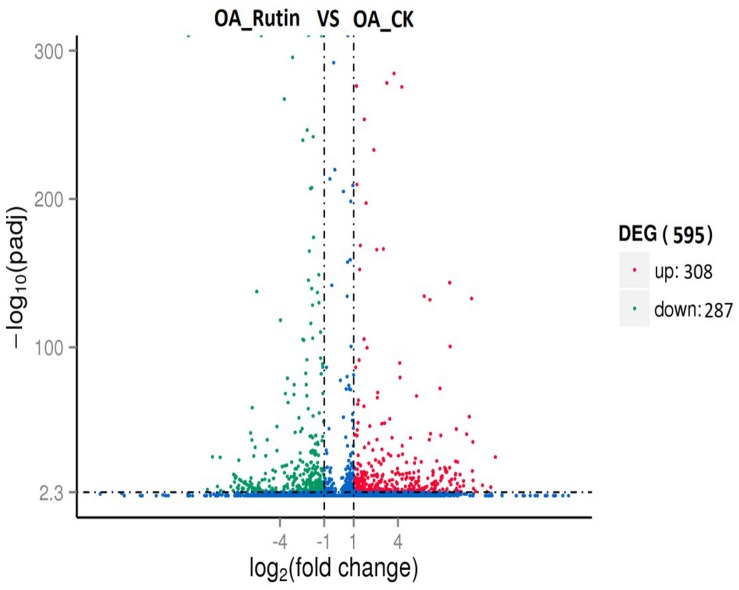
Differentially expressed genes analysis (adjusted *P* < 0.05, | log_2_.Fold_change| > 1) between *O. asiaticus* feeding on rutin-treated foods (OA_Rutin) and no rutin-treated foods (OA_CK). Genes were divided among three classes: red genes are significantly upregulated in the right sample versus the left sample; green genes are significantly downregulated in the right sample versus the left sample; and blue genes are not significantly differentially expressed.

Differentially expressed genes (adjusted *P*<0.01, | log_2_.Fold_change|>1) between the control and rutin-challenged libraries were identified. Compared with controls, *O. asiaticus* feeding on rutin had 308 upregulated genes and 287 downregulated genes (Figure 2 and Supplementary Tables S5, S6).

Among the upregulated genes (Supplementary Table S5), many stress-resistant or detoxifying enzymes were identified, including cytochrome P450 9E1, heat shock protein, cytochrome P450 6k1, beta-glucosidase, catalase, carboxylesterase, peroxidase, superoxide dismutase, UDP-glucuronosyltransferase, and lactase-phlorizin hydrolase. This suggested that feeding on rutin could induce the expression of stress-resistance- and detoxification-related genes in the grasshopper *O. asiaticus*.

The downregulated genes mainly belonged to insect cuticle biosynthesis, development, and nutrition metabolism; these included vitellogenin, larval cuticle protein 2, chitin synthase 1 variant B, cuticle protein 6, hexokinase type 2, and lipase 3 (Supplementary Table S6).

### GO and KEGG Pathway Enrichment Analysis of DEGs

**FIGURE 3:**
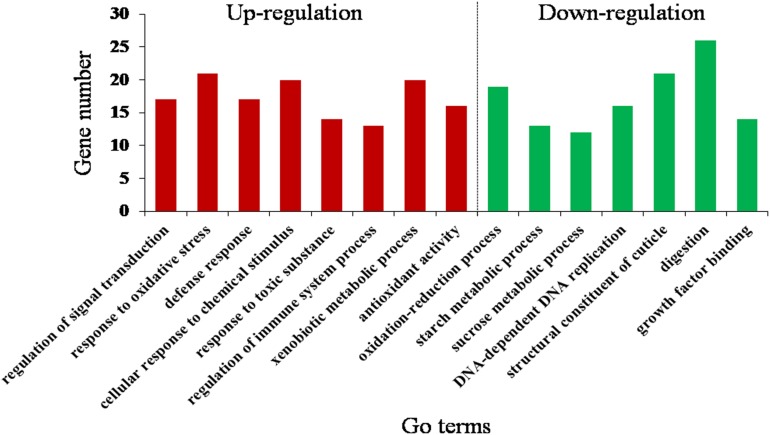
Gene Ontology (GO) enrichment analysis (corrected *P* < 0.05) of the differentially expressed genes of *O. asiaticus* fed on rutin-treated food compared to those fed on no rutin-treated food. The *y*-axis indicates the number of genes in each Go term.

By the GOseq R package, the DEGs were mainly assigned to 15 GO terms (corrected *P* < 0.05) (Figure 3 and Supplementary Table S7). Downregulated GO terms included oxidation–reduction process, starch metabolic process, sucrose metabolic process, DNA-dependent DNA replication, structural constituent of cuticle, digestion, and growth factor binding. Upregulated GO terms included response to oxidative stress, regulation of signal transduction, defense response, response to toxic substance, regulation of immune system process, xenobiotic metabolic process and antioxidant activity, and cellular response to chemical stimulus. This suggests that *O. asiaticus* feeding on xenobiotic rutin had increased signal transduction, stress resistance, and metabolizing capacity of toxic substances.

**FIGURE 4:**
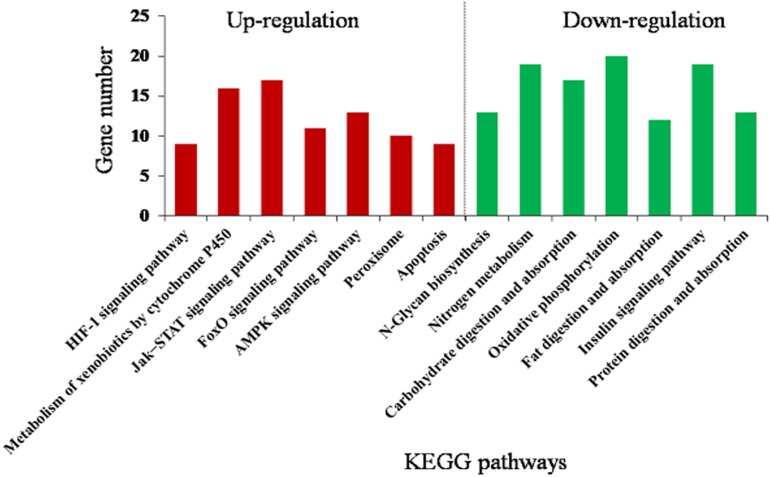
Kyoto Encyclopedia of Genes and Genomes (KEGG) enrichment analysis (corrected *P* < 0.05) of the differentially expressed genes in *O. asiaticus* fed on rutin-treated food compared to those fed on no rutin-treated food. The *y*-axis indicates the number of genes in each KEGG pathway.

The DEGs were mainly assigned to 14 (corrected *P* < 0.05) pathways (Figure 4 and Supplementary Table S8). Downregulated pathways included N-glycan biosynthesis, nitrogen metabolism, carbohydrate digestion and absorption, oxidative phosphorylation, fat digestion and absorption, insulin signaling pathway, and protein digestion and absorption. Upregulated pathways included metabolism of xenobiotics by cytochrome P450, hypoxia-inducible factor 1 (HIF-1) signaling pathway, FoxO signaling pathway, Jak–STAT signaling pathway, AMP-activated protein kinase (AMPK) signaling pathway, peroxisome, and apoptosis. This suggests that rutin-fed *O. asiaticus* had increased activities in detoxification, immune response, and stress resistance.

### Verification of the Gene Expression Through qRT-PCR

**FIGURE 5:**
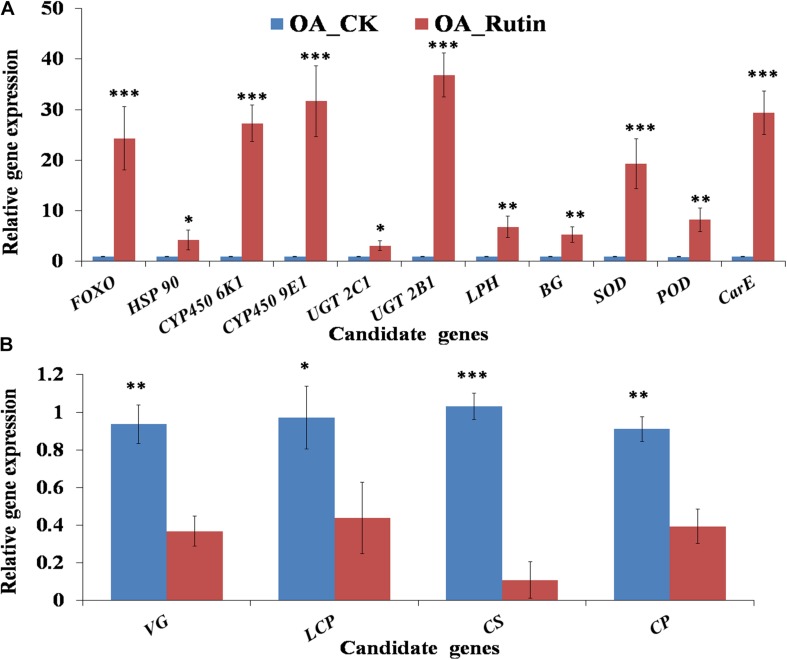
Quantitative real-time PCR (qRT-PCR) analysis of 15 candidate genes. **(A)** Significantly up-regulated candidate genes in grasshoppers fed on rutin, **(B)** Significantly down-regulated candidate genes in grasshoppers fed on rutin. The results were evaluated using the 2^–Δ Δ CT^ method. The 2^–Δ Δ CT^ values of controls were set to one to calibrate the relative gene expression levels. Bars represent mean ± SD values. ^∗^*P* < 0.05, ^∗∗^*P* < 0.01, ^∗∗∗^*P* < 0.001 (Student’s *t* test).

Relative gene expression of 15 candidate genes were tested by qRT-PCR. Results showed that the metabolism- or cuticle biosynthesis-related genes *VG*, *LCP*, *CS*, and *CP* were significantly downregulated in *O. asiaticus* fed on rutin (*P* < 0.05, Figure 5). In contrast, the stress-resistance- or detoxification-related genes *FOXO*, *HSP 90*, *CYP450 6K1*, *CYP450 9E1*, *UGT 2C1*, *UGT 2B1*, *LPH*, *BG*, *SOD*, *POD*, and *CarE* were significantly upregulated (*P* < 0.05, Figure 5). Moreover, 15 genes showed significant correlations (*R*^2^ = 0.8937, *P* < 0.05) between the qRT-PCR data and the RNA-seq results, which indicated good reproducibility between transcript abundance assayed by RNA-seq and the expression profile revealed by the qRT-PCR data.

## Discussion

In our experiment, we employed a feeding trial and RNA-seq to compare the suitability of xenobiotic rutin for the grasshopper *O. asiaticus*. The results demonstrate that xenobiotic rutin, as a phytotoxin, is detrimental for *O. asiaticus*. Grasshoppers fed on rutin-treated food had reduced size, growth, and survival rate. These results were consistent with those from previous research concerning the effects of flavonoid rutin on other insects (Simmonds, 2003; Taggar and Gill, 2016; Chen et al., 2017) and supported the hypothesis that insect growth performance was negatively correlated with plant toxic compounds. Subsequent transcriptomic analysis demonstrated that rutin-fed grasshoppers exhibited dramatically different transcription profiles from control grasshoppers. Why would feeding on rutin alter transcription profiles, and what are the consequences of such changes? These are all important questions that need to be addressed.

Understanding the genetic and molecular basis of insect response mechanisms to chemical pressures (e.g., plant allelochemicals and other toxic compounds) is a key challenge in developing new pest control strategies. Toxic compounds in environment pose a constant challenge to the survival of insect pests (Després et al., 2007; Caballero et al., 2008; Chen et al., 2017). These toxins originating from a wide range of sources, such as plant toxins or chemical pesticides, generally have detrimental effects on insect pests (Senthil-Nathan, 2013; Birnbaum et al., 2017). To avoid these poisonous effects and sustain survival and reproduction, insects have evolved multiple strategies to overcome these chemical toxins, mainly involving the complex induction of transporters, digestion, detoxification, or immune-related genes or pathways (Misra et al., 2011; Senthil-Nathan, 2013; Herde and Howe, 2014; Roy et al., 2016). In the present study, we found that these related genes and pathways were also significantly changed in grasshoppers feeding on rutin. Rutin induced 308 upregulated and 287 downregulated genes in *O. asiaticus*. Rutin-fed grasshoppers had increased activities in detoxification, immune, and stress resistance, and had decreased biosynthesis and nutrition metabolism.

Research over the past several decades has shown that the molecular adaptations of herbivorous insects to plant toxins mainly involve toxin degradation (metabolic adaptation) and target-site mutations (Salunke et al., 2005; Sun et al., 2006; Misra et al., 2011). Generally, metabolic adaptations of insects, in the most common strategy via biotransformation of plant toxins, include phases I, II, and III mechanisms (Després et al., 2007; Birnbaum and Abbot, 2018). These three metabolic mechanisms are involved in many detoxification enzymes, such as cytochrome P450s, carboxylesterases, glutathione-*S*-transferases (GSTs), and uridine diphosphate (UDP)-glycosyltransferases (UGTs). Through these complex processes, insects can modify the ingested chemical toxins and render them less toxic and easier to transport or excrete (Chahine and O’Donnell, 2011; Dobler et al., 2011). In the present study, grasshoppers feeding on rutin also had significantly upregulated gene expression for cytochrome P450 6k1, cytochrome P450 9E1, carboxylesterase, UDP-glucuronosyltransferase 2B1, UDP-glucuronosyltransferase 2C1, and glutathione-*S*-transferases. These detoxification enzymes of grasshoppers may be significantly activated to enhance their catalytic activity toward toxic rutin. Aside from metabolic adaptations, many insect species have evolved “target-site modifications” at the binding location of toxins to protect themselves from plant toxic compounds (Després et al., 2007; Zhen et al., 2012; Ujvari et al., 2015). In addition, some gut microbes in insects can play important roles in the detoxification of toxic compounds (Henry et al., 2013; Giron et al., 2017; Birnbaum and Abbot, 2018). However, whether these mechanisms were also involved in the resistance of grasshoppers to rutin should be studied in the future.

In regard to the other specific DEGs for qRT-PCR, feeding on rutin resulted in downregulation of vitellogenin, larval cuticle protein, chitin synthase, and cuticle protein. These genes are related to insect development and cuticle biosynthesis. The upregulation of genes included heat shock protein, FoxO protein, peroxidase, and superoxide dismutase. These upregulated genes are related with insect stress resistance and oxidation–reduction and well known to be highly inducible and to ameliorate stress (Hwangbo et al., 2004; Krishnan and Kodrík, 2006; Lehtinen et al., 2006; Després et al., 2007; Tang et al., 2013). In addition, the upregulation of these genes is clearly beneficial for grasshoppers’ survival. In contrast, we do not know if the downregulated genes are detrimental, beneficial, or simply accidental by-products of rutin stress. These rapid biochemical responses to toxic rutin may be vital for insect survival and growth. The reasons why these genes were downregulated need further study.

We acknowledge that, although the potential functions of some DEGs were discussed in the present study, more lab evidences should be provided to characterize their roles in response to rutin in the future. For example, we found that the gene expression levels of beta-glucosidase and lactase-phlorizin hydrolase were significantly upregulated. In mammals, these two enzymes can participate in the hydrolysis process of flavonoids by cutting off the glucosidic bond (Poreddy et al., 2015; Kohl et al., 2016). If these two hydrolases also participated in the hydrolysis of rutin in grasshoppers, it could be studied by gene technology (e.g., HIF-1) in the future.

From the GO and KEGG enrichment of DEGs, we found that the structural constituents to cuticle production and nutrition metabolic processes were significantly downregulated, and the pathways related to detoxification, stress resistance, and immunity (e.g., the Jak-STAT signaling pathway, metabolism of xenobiotics by cytochrome P450, and the FoxO signaling pathway) were significantly upregulated in rutin-fed grasshoppers. The fact that *O. asiaticus* upregulated stress resistance genes or pathways after feeding on rutin is not surprising, given that rutin is a toxic compound (Salunke et al., 2005; Kramer et al., 2008; Chen et al., 2017).

The altered transcriptome offers insights into the observed growth performance of grasshoppers exposed to rutin. Changes in phenotype indicate that the grasshoppers exhibit phenotypic plasticity when confronted with toxic rutin. Any organism can undergo such phenotypic plasticity, which can be expressed as changes in morphology or physiology (West-Eberhard, 2003; Whitman and Ananthrakrishnan, 2009). Many of these changes are probably responses to toxic stress, as indicated by the significantly lower growth performance observed in this study. Indeed, transcription underlies most phenotypic plasticity (Valentino and Harrelson, 2013). Small changes in transcription generally can produce significant changes to phenotypes. In this study, the changed transcription represented grasshoppers’ phenotypic plasticity because it altered the phenotype (growth performance). Altered transcription responding to environmental stress can range from highly evolved and beneficial responses to non-evolved responses and consequently may have beneficial, neutral, or detrimental effects to living organisms (Whitman and Ananthrakrishnan, 2009; Valentino and Harrelson, 2013). Sorting out those effects from altered transcription is difficult. For example, transcriptomic changes of a single enzyme may influence numerous other enzymes or molecular pathways and consequently alter numerous divergent morphological and physiological aspects. Some of these changes may be beneficial, while others may produce detrimental effects (DeWitt and Scheiner, 2004; Whitman and Ananthrakrishnan, 2009). An example is the transcriptional changes delaying growth or reproduction; these may at first appear to be detrimental but in fact may be beneficial if they allow the individual to survive during a period of stress, such as during poisoning in the present study. In addition, the molecular or physiological mechanisms insects use to detoxify toxins as described above are generally assumed to be costly (Després et al., 2007). Costs of toxin resistance, such as reduced survival, fecundity, or energy reserves, have been demonstrated in many insect species (Rivero et al., 2011; Gordon et al., 2015; Schwenke et al., 2016). In the present study, grasshoppers feeding on rutin had higher levels of gene expression associated with detoxification or immunity, which implies that grasshopper survival requires greater consumption to detoxify rutin and consequently results in reduced phenotypic parameters such as size and growth rate compared to grasshoppers feeding on untreated foods.

One broad conclusion can be drawn from this study. Plant toxic compounds induce changed gene expression profiles of herbivorous insects. This confirms the hypothesis that altered transcription is related to environmental stress (Enders et al., 2015; Roy et al., 2016). Toxic stress substantially alters insect gene expression. In the present study, rutin-fed grasshopper exhibited 595 DEGs in comparison to untreated grasshoppers. In our case, we know that grasshoppers feeding on rutin were stressed because the feeding significantly lowered insect performance.

We explored phenotypic and transcriptomic responses in herbivores to toxic rutin using “Omics” technologies and found that rutin had detrimental effects to grasshoppers. This can offer future opportunities in the development of rutin-related insecticides for grasshopper control. *O. asiaticus*, as an important grassland pest, can cause severe grassland damage and lead to large economic losses in northern China (Cease et al., 2012). Traditional control of this grasshopper species relies too much on synthetic pesticides. To help reduce the occurrence of this disaster in an ecofriendly manner, the use of botanical pesticides is of great significance. In the future, we can develop and apply rutin-related insecticides or use rutin as the adjuvant to achieve new breakthroughs in grasshopper control.

## Data Availability Statement

The datasets generated for this study can be found in the NCBI, accession number SRP072969.

## Ethics Statement

Grasshoppers *Oedaleus asiaticus* are common agricultural pests and are not included in the “List of Protected Animals in China.” No specific permits were required for the described studies.

## Author Contributions

XH designed the experiments. XH, SL, and BC performed the experiments and wrote the manuscript. XH, BC, and ZZ analyzed the data. All authors reviewed and considered the manuscript.

## Conflict of Interest

The authors declare that the research was conducted in the absence of any commercial or financial relationships that could be construed as a potential conflict of interest.

## Footnotes

1 http://bioinfo.ut.ee/primer3-0.4.0/
