# Supplementary material for: Phenotypic and Transcriptomic Response of the Grasshopper Oedaleus asiaticus (Orthoptera: Acrididae) to Toxic Rutin
Source: Front Physiol. 2020 Feb 21;11:52. doi: 10.3389/fphys.2020.00052 (PMC7047750; doi:10.3389/fphys.2020.00052)
Supplement: Supplementary file 1 [file Data_Sheet_1.PDF]

Table S1 The selected DEGs for qRT-PCR analysis

Gene ID	Description	Abbreviation	Fold change OA_Rutin VS. OA_CK
Cluster-11327.75652	FoxO protein	*FOXO*	8.3962
Cluster-11327.59722	heat shock protein 90	*HSP 90*	1.2231
Cluster-11327.54882	cytochrome P450 6 K1	*CYP450 6K1*	6.3763
Cluster-11327.70406	cytochrome P450 9E1	*CYP450 9E1*	6.8194
Cluster-11327.22262	UDP-glucuronosyltransferase 2C1	*UGT 2C1*	1.2448
Cluster-11327.24983	UDP-glucuronosyltransferase 2B1	*UGT 2B1*	8.9884
Cluster-11327.46783	Lactase-phlorizin hydrolase	*LPH*	2.6234
Cluster-11327.3892	beta-glucosidase	*BG*	1.8551
Cluster-11327.55763	superoxide dismutase	*SOD*	7.6322
Cluster-11327.56874	Peroxidase	*POD*	1.9214
Cluster-11327.66426	carboxylesterase	*CarE*	9.7385
Cluster-11327.56459	vitellogenin	*VG*	-5.6797
Cluster-11327.87505	Larval cuticle protein 2	*LCP*	-1.5600
Cluster-11327.80081	Chitin synthase 1	*CS*	-5.4903
Cluster-11327.93652	Cuticle protein 7	*CP*	-4.8046

Table S2 Designed sequences of qRT-PCR primers for 16 genes

Gene discription	Sequence of primers (5' to 3')	
cytochromeP450 9E1 *CYP450 9E1*	Forward	ACGAGAGAGAAGCAGGGGAT
	Reverse	AGCAGCAGAAGCACGTAACT
Carboxylesterase *CarE*	Forward	CGACTTGGGACGTTAGGCTT
	Reverse	AAGAAATGAGGAGGGCGTGG
superoxide dismutase *SOD*	Forward	CCAACAGCAGGACTTCGCTA
	Reverse	CACAGTTCTCTGGCTGAGGG
UDP-glucuronosyltransferase 2C1 *UGT 2C1*	Forward	CTACATGGTGAGCGAGCGAT
	Reverse	GGAGGCGTGTGGAGAGAAAA
Peroxidase *POD*	Forward	GCTGAATGGTGTCTGGGTCA
	Reverse	GAAGCGACGCAGCTTATTGG
heat shock protein 90 *HSP 90*	Forward	CCAATCTGGACCAGGAACCC
	Reverse	TCCAGATGCTGATGTTGCGT
beta-glucosidase *BG*	Forward	AGGAACTGGGGGCAAAGATG
	Reverse	GCTCGTACCAGAAGCTGTGT
FoxO protein *FOXO*	Forward	CATCACGCAGGCCATCCAG
	Reverse	GCTGTTGCTGTCGCCCTTG
Lactase-phlorizin hydrolase *LPH*	Forward	GTGAGGTGGTGGATCACGTT
	Reverse	CACCTTCCCTTTTTGCGTGG
UDP-glucuronosyltransferase 2B1	Forward	AGCTTCTCAGTTCGCTGCTT

UGT 2B1	Reverse	TTTAACACGCAAGTGGCTGC
cytochrome P450 6 K1<br>*CYP450 6K1*	Forward<br>Reverse	GGCATTGTCGGTCCAAAACC<br>TCGAACGTCCCGAAAACCTT
vitellogenin<br>*VG*	Forward<br>Reverse	CGCAACGCCACTATTAAGGC<br>GATACGACGTCCTCACCGAC
Larval cuticle protein 2<br>*LCP*	Forward<br>Reverse	CCACATAGCTGTAGGAGCCG<br>ACAACATCGTTTTGGTGGCG
Chitin synthase 1<br>*CS*	Forward<br>Reverse	TACGCAAGTCTCGGCTGTTT<br>AGAAACCTGAGCACAGACGG
Cuticle protein 7<br>*CP*	Forward<br>Reverse	GTACGTCAGCGGGGACAC<br>GTCCACCACAAACACACCAT
β-actin	Forward<br>Reverse	CCCATCTATGAAGGTTACGC<br>CTTGATGTCACGGACGATTT

Table S3. Summary of RNA-seq metrics from *O. asiaticus* transcriptomes. Key: OA_CK (*O. asiaticus* feeding on foods of CK), OA_Rutin (*O. asiaticus* feeding on food treated with rutin), respectively.

Sample	Raw Reads	Clean reads	Error rate(%)	Q20(%)	Q30(%)	GC(%)
OA_CK1	85240756	81766900	0.02	96.62	91.85	45.89
OA_CK2	113348312	108908962	0.02	96.71	91.98	47.92
OA_CK3	96353524	92036266	0.02	96.17	91.02	47.66
OA_Rutin 1	87612662	84134408	0.02	96.64	91.88	46.21
OA_Rutin 2	91177710	87538360	0.02	96.88	92.37	46.45
OA_Rutin 3	88,691,840	86648397	0.02	95.29	90.32	45.93

Table S4 Annotation results of unigenes

	Number of Unigenes	Percentage (%)
Annotated in NR	54162	40.67
Annotated in NT	17040	12.79
Annotated in KEGG	19392	14.56
Annotated in SwissProt	37473	28.14
Annotated in PFAM	45647	34.28
Annotated in GO	45846	34.43
Annotated in KOG	26428	19.84

Annotated in all Databases	7531	5.65
Annotated in at least one Database	67101	50.39
Total Unigenes	133144	100

Table S5 The up-regulated genes (adjusted *P* values < 0.05, $|\log_2.\text{Fold\_change}| > 1$) of *O. asiaticus* feeding rutin-treated foods compared with individuals feeding no rutin-treated foods.

Gene ID	Description	Fold change
Cluster-18495.1	PREDICTED: uncharacterized protein LOC103519961, partial [Diaphorina citri]	7.8617
Cluster-11327.22702	PREDICTED: zinc finger BED domain-containing protein 1-like [Plutella xylostella]	5.5797
Cluster-11327.57127	cathepsin L [Riptortus pedestris]	6.0653
Cluster-11327.51551	Translocation protein SEC62 [Zootermopsis nevadensis]	5.4187
Cluster-11327.59757	Muscle M-line assembly protein unc-89 [Zootermopsis nevadensis]	3.7802
Cluster-11327.53165	coracle, partial [Blattella germanica]	6.3911
Cluster-11327.23836	Nucleolar protein 6 [Zootermopsis nevadensis]	6.5367
Cluster-11327.57341	hypothetical protein L798_15728, partial [Zootermopsis nevadensis]	5.3966
Cluster-11327.54319	PREDICTED: nardilysin-like [Athalia rosae]	7.3238
Cluster-22001.0	hypothetical protein YQE_02226, partial [Dendroctonus ponderosae]	6.851
Cluster-11327.86553	HR3 isoform A [Blattella germanica]	7.8645
Cluster-11327.65652	PREDICTED: tyrosine-protein phosphatase Lar isoform X9 [Acromyrmex echinatior]	5.2048
Cluster-11327.57657	PREDICTED: stearoyl-CoA desaturase 5 [Musca domestica]	6.3647
Cluster-11327.53820	PREDICTED: plexin-B [Megachile rotundata]	6.7596
Cluster-11327.57183	Lysosomal aspartic protease [Zootermopsis nevadensis]	6.5521
Cluster-11327.2863	histidine acid phosphatase domain containing 2A [Capsaspora owczarzaki ATCC 30864]	8.8357
Cluster-11327.38973	Vacuolar fusion protein MON1-like protein A [Zootermopsis nevadensis]	9.1288
Cluster-11327.55212	coracle, partial [Blattella germanica]	6.8194
Cluster-11327.61598	Titin [Zootermopsis nevadensis]	6.1134
Cluster-11327.22601	arrestin1 precursor [Dianemobius nigrofasciatus]	9.7385
Cluster-11327.40539	Nucleolar protein 6 [Zootermopsis nevadensis]	2.4975
Cluster-11327.51896	phosphoenolpyruvate carboxykinase [Blattella germanica]	7.9736
Cluster-11327.58243	alpha tubulin [Schistocerca gregaria]	7.2293
Cluster-11327.11455	PREDICTED: RNA-directed DNA polymerase from mobile element jockey-like, partial [Diaphorina citri]	8.03
Cluster-11327.41297	Autophagy protein 5 [Zootermopsis nevadensis]	8.4514
Cluster-10711.0	Calcium-dependent secretion activator [Zootermopsis nevadensis]	7.2528
Cluster-11327.75660	hypothetical protein L798_13582 [Zootermopsis nevadensis]	7.8454

Cluster-11327.57299	cathepsin L [Riptortus pedestris]	5.3105
Cluster-11327.57771	Hexokinase type 2 [Zootermopsis nevadensis]	5.6199
Cluster-11327.56873	Juvenile hormone epoxide hydrolase 1 [Zootermopsis nevadensis]	3.4232
Cluster-11327.88658	Vesicular acetylcholine transporter [Zootermopsis nevadensis]	4.6943
Cluster-11327.54429	Tuberin [Zootermopsis nevadensis]	4.9269
Cluster-11327.75537	hypothetical protein L798_07553, partial [Zootermopsis nevadensis]	7.6322
Cluster-11327.70732	hypothetical protein L798_07553, partial [Zootermopsis nevadensis]	9.2654
Cluster-11327.84554	AGAP007928-PA-like protein [Anopheles sinensis]	3.6205
Cluster-11327.62388	PREDICTED: muscle M-line assembly protein unc-89 isoform X2 [Monomorium pharaonis]	4.9342
Cluster-11327.60687	Putative cysteine proteinase [Zootermopsis nevadensis]	1.4836
Cluster-11327.53657	RAC serine/threonine-protein kinase [Zootermopsis nevadensis]	5.0747
Cluster-11327.16133	Ribonuclease H2 subunit A [Zootermopsis nevadensis]	3.7825
Cluster-11327.63188	coracle, partial [Blattella germanica]	6.2115
Cluster-11327.68541	PREDICTED: sodium-dependent nutrient amino acid transporter 1 [Acyrthosiphon pisum]	1.1902
Cluster-11327.56563	Fibronectin type-III domain-containing protein 3a [Zootermopsis nevadensis]	2.1158
Cluster-11327.59522	5'-AMP-activated protein kinase subunit gamma-2 [Harpegnathos saltator]	6.5575
Cluster-11327.58089	PREDICTED: filamin-A isoform X1 [Tribolium castaneum]	5.3717
Cluster-11327.80566	PREDICTED: hepatocyte nuclear factor 4-gamma isoform X5 [Tribolium castaneum]	4.2574
Cluster-11327.91178	PREDICTED: regulator of telomere elongation helicase 1 homolog [Athalia rosae]	4.5053
Cluster-11327.62901	PREDICTED: fatty acid synthase [Acyrthosiphon pisum]	1.441
Cluster-11327.56867	Solute carrier family 2, facilitated glucose transporter member 3 [Zootermopsis nevadensis]	5.0798
Cluster-11327.61551	Putative cysteine proteinase [Zootermopsis nevadensis]	1.562
Cluster-11327.63962	Putative epidermal cell surface receptor [Zootermopsis nevadensis]	2.024
Cluster-11327.66216	PREDICTED: protein turtle isoform X3 [Tribolium castaneum]	2.692
Cluster-11327.49228	RXR [Locusta migratoria]	4.6097
Cluster-11327.70970	RAC serine/threonine-protein kinase [Zootermopsis nevadensis]	3.9768
Cluster-11327.82592	PREDICTED: transcription factor HNF-4 homolog isoform X9 [Tribolium castaneum]	5.2708
Cluster-11327.62955	Lipin-3 [Zootermopsis nevadensis]	5.157
Cluster-11327.16601	argonaute-3 [Nilaparvata lugens]	5.4105
Cluster-18023.1	PREDICTED: uncharacterized protein K02A2.6-like [Vollenhovia emeryi]	5.0053

Cluster-11327.28595	hypothetical protein L798_00578 [Zootermopsis nevadensis]	2.8911
Cluster-11327.46205	hypothetical protein L798_00578 [Zootermopsis nevadensis]	2.0607
Cluster-11327.78214	Multidrug resistance-associated protein 7 [Zootermopsis nevadensis]	5.0701
Cluster-11327.56888	heat shock protein 20.6 [Locusta migratoria]	2.8316
Cluster-11327.54368	Tuberin [Zootermopsis nevadensis]	3.3049
Cluster-11327.69481	putative helicase with zinc finger domain [Zootermopsis nevadensis]	6.3742
Cluster-11327.27731	Ankyrin repeat and fibronectin type-III domain-containing protein 1, partial [Zootermopsis nevadensis]	5.057
Cluster-11327.59047	Hexokinase type 2 [Zootermopsis nevadensis]	1.1493
Cluster-11327.82860	Nucleolar protein 6 [Zootermopsis nevadensis]	2.0375
Cluster-11327.2616	PREDICTED: Down syndrome cell adhesion molecule-like protein Dscam2 [Athalia rosae]	5.1318
Cluster-11327.98782	hypothetical protein L798_04980 [Zootermopsis nevadensis]	2.2247
Cluster-11327.65161	E3 ubiquitin-protein ligase arkadia-B [Zootermopsis nevadensis]	2.602
Cluster-11327.74072	Insulin receptor [Zootermopsis nevadensis]	2.859
Cluster-11327.53673	PREDICTED: uncharacterized protein LOC656511 [Tribolium castaneum]	3.9717
Cluster-11327.56209	PREDICTED: twitchin [Atta cephalotes]	2.5463
Cluster-11327.40540	Nucleolar protein 6 [Zootermopsis nevadensis]	4.7815
Cluster-11327.47601	Iron/zinc purple acid phosphatase-like protein [Zootermopsis nevadensis]	2.9272
Cluster-11327.66717	Calcium/calmodulin-dependent protein kinase kinase 2 [Zootermopsis nevadensis]	5.4036
Cluster-11327.87684	PREDICTED: putative inorganic phosphate cotransporter [Musca domestica]	2.2913
Cluster-11327.12998	PREDICTED: protein turtle isoform X7 [Tribolium castaneum]	4.9028
Cluster-11327.56358	Mitochondrial ornithine transporter 1 [Zootermopsis nevadensis]	1.2176
Cluster-11327.36721	RAD50-interacting protein 1 [Zootermopsis nevadensis]	2.6472
Cluster-11327.83896	GABA-gated chloride channel subunit [Sogatella furcifera]	5.0411
Cluster-11327.45657	PREDICTED: neurotrimin-like isoform X2 [Tribolium castaneum]	1.4114
Cluster-11327.65480	Sestrin-like protein [Zootermopsis nevadensis]	6.4573
Cluster-11327.57502	hypothetical protein L798_03331, partial [Zootermopsis nevadensis]	4.2889
Cluster-11327.48523	Ankyrin repeat and fibronectin type-III domain-containing protein 1, partial [Zootermopsis nevadensis]	2.63
Cluster-11327.59242	Acyl-CoA Delta(11) desaturase [Zootermopsis nevadensis]	1.227
Cluster-11327.56301	Cytokine receptor [Zootermopsis nevadensis]	7.5088
Cluster-11327.100954	PREDICTED: steroid 17-alpha-hydroxylase/17,20 lyase-like [Poecilia reticulata]	2.9212

Cluster-11327.90804	predicted protein [Nematostella vectensis]	3.0207
Cluster-11327.97183	Fasciclin-2 precursor, putative [Pediculus humanus corporis]	1.5683
Cluster-11327.56141	heat shock protein 20.6 [Locusta migratoria]	6.3974
Cluster-11327.51895	phosphoenolpyruvate carboxykinase [Blattella germanica]	3.2706
Cluster-25000.0	thioredoxin reductase 2, variant [Salpingoeca rosetta]	1.6787
Cluster-10359.0	PREDICTED: uncharacterized protein LOC105283414 [Cerapachys biroi]	5.8171
Cluster-11327.40796	Ankyrin repeat and fibronectin type-III domain-containing protein 1, partial [Zootermopsis nevadensis]	2.2574
Cluster-11327.55700	Carnitine O-palmitoyltransferase 1, liver isoform [Zootermopsis nevadensis]	1.4166
Cluster-11327.59096	GAPDH [Locusta migratoria]	5.918
Cluster-11327.56629	hypothetical protein TcasGA2_TC004196 [Tribolium castaneum]	7.0024
Cluster-11327.56459	vitellogenin [Cyrtorhinus lividipennis]	7.911
Cluster-11327.53067	Tenascin-X [Zootermopsis nevadensis]	2.9846
Cluster-11327.63689	Microsomal triglyceride transfer protein large subunit [Zootermopsis nevadensis]	9.0995
Cluster-11327.59161	PREDICTED: carnitine O-acetyltransferase-like, partial [Plutella xylostella]	9.0536
Cluster-11327.55961	Translation initiation factor IF-2 [Cricetulus griseus]	4.0009
Cluster-11327.56438	Protein hu-li tai shao [Zootermopsis nevadensis]	3.8374
Cluster-11327.46754	PREDICTED: aminopeptidase N [Pogonomyrmex barbatus]	1.1665
Cluster-11327.57432	PREDICTED: uncharacterized protein LOC105360525 [Ceratosolen solmsi marchali]	1.1256
Cluster-11327.56020	PREDICTED: flexible cuticle protein 12 [Tribolium castaneum]	1.4563
Cluster-11327.56022	Electron transfer flavoprotein subunit beta [Zootermopsis nevadensis]	5.0496
Cluster-11327.48398	PREDICTED: dynamin-like 120 kDa protein, mitochondrial isoform X1 [Athalia rosae]	7.2639
Cluster-11327.58041	C3 and PZP-like alpha-2-macroglobulin domain-containing protein 8 [Zootermopsis nevadensis]	8.4484
Cluster-11327.53166	hypothetical protein L798_07481 [Zootermopsis nevadensis]	7.9178
Cluster-11327.56485	PREDICTED: muscle M-line assembly protein unc-89 [Athalia rosae]	5.0755
Cluster-11327.52447	chemosensory protein [Chilo auricilius]	4.5341
Cluster-11327.57341	hypothetical protein L798_15728, partial [Zootermopsis nevadensis]	2.3213
Cluster-11327.57178	LIM domain and actin-binding protein 1 [Zootermopsis nevadensis]	1.2989
Cluster-11327.56226	Cytochrome b-c1 complex subunit 2, mitochondrial [Zootermopsis nevadensis]	1.5282
Cluster-11327.51794	PREDICTED: oxidation resistance protein 1 isoform X7 [Monomorium pharaonis]	2.0632

Cluster-11327.62704	PREDICTED: acylphosphatase-1-like [Apis florea]	2.031
Cluster-11327.48215	Collagen alpha-1(XI) chain, partial [Zootermopsis nevadensis]	3.9685
Cluster-11327.53136	PREDICTED: delta-1-pyrroline-5-carboxylate dehydrogenase, mitochondrial isoform X2 [Acyrthosiphon pisum]	5.0499
Cluster-11327.56041	PREDICTED: muscle-specific protein 20-like [Megachile rotundata]	5.5253
Cluster-11327.56048	40S ribosomal protein S5a [Zootermopsis nevadensis]	1.8353
Cluster-11327.53645	arylphorin hexamerin-like protein 2 [Romalea microptera]	7.1714
Cluster-11327.51481	RecName: Full=Rhodopsin; Short=Opsin [Sphodromantis sp.]	5.3381
Cluster-11327.67900	Mite allergen Lep d 7 [Zootermopsis nevadensis]	1.2951
Cluster-11327.55944	PREDICTED: titin isoform X3 [Bombus terrestris]	3.0884
Cluster-11327.46428	PREDICTED: protein Malvolio isoform X2 [Microplitis demolitor]	1.0604
Cluster-11327.63404	60s ribosomal protein l7 [Lasius niger]	1.1319
Cluster-11327.58073	Carnitine O-palmitoyltransferase 2, mitochondrial [Zootermopsis nevadensis]	1.1638
Cluster-11327.55584	60S ribosomal protein L12 [Zootermopsis nevadensis]	1.3218
Cluster-11327.56690	Aspartate aminotransferase, mitochondrial [Zootermopsis nevadensis]	1.0601
Cluster-11327.61337	hypothetical protein KGM_12267 [Danaus plexippus]	1.2287
Cluster-11327.60785	uncharacterized protein [Coptotermes formosanus]	2.8536
Cluster-11327.50627	hypothetical protein YQE_04947, partial [Dendroctonus ponderosae]	1.0234
Cluster-11327.57908	laminin A chain, putative [Pediculus humanus corporis]	2.0916
Cluster-11327.54515	Ligand of Numb protein X 2 [Zootermopsis nevadensis]	2.4766
Cluster-11327.56584	PREDICTED: malate dehydrogenase, cytoplasmic [Musca domestica]	1.7062
Cluster-11327.72662	endonuclease-reverse transcriptase [Eyprepocnemis plorans plorans]	5.0217
Cluster-11327.52547	PREDICTED: pyridoxal kinase [Tribolium castaneum]	3.9845
Cluster-11327.48958	PREDICTED: ras and Rab interactor 1 [Danio rerio]	1.1067
Cluster-11327.51972	hypothetical protein L798_04814 [Zootermopsis nevadensis]	1.2433
Cluster-11327.59293	RecName: Full=Endocuticle structural glycoprotein ABD-4 [Locusta migratoria]	3.0027
Cluster-11327.57470	Dehydrogenase/reductase SDR family member 11 [Zootermopsis nevadensis]	5.9652
Cluster-11327.70020	hypothetical protein TcasGA2_TC006727 [Tribolium castaneum]	4.5747
Cluster-11327.61361	PREDICTED: uncharacterized protein	3.6187

	LOC105556100 [Vollenhovia emeryi]	
Cluster-11327.61362	PREDICTED: uncharacterized protein LOC105556100 [Vollenhovia emeryi]	5.4869
Cluster-11327.62606	putative adenylate kinase isoenzyme F38B2.4 [Zootermopsis nevadensis]	1.2001
Cluster-11327.49642	enzymatic polyprotein, putative [Pediculus humanus corporis]>gi|212516795|gb|EEB18763.1| enzymatic polyprotein, putative [Pediculus humanus corporis]	1.0215
Cluster-11327.57957	Endocuticle structural glycoprotein SgAbd-2, partial [Zootermopsis nevadensis]	4.9412
Cluster-11327.59905	hypothetical protein L798_14756 [Zootermopsis nevadensis]	1.9681
Cluster-11327.58542	actin-depolymerizing factor 1 [Coptotermes formosanus]	1.8756
Cluster-11327.60010	PREDICTED: PDZ and LIM domain protein Zasp-like isoform X3 [Megachile rotundata]	2.4817
Cluster-11327.61768	PREDICTED: NAD kinase-like isoform X10 [Diaphorina citri]	1.0975
Cluster-11327.52948	obstructor A1 [Locusta migratoria]	3.4429
Cluster-11327.62388	PREDICTED: muscle M-line assembly protein unc-89 isoform X2 [Monomorium pharaonis]	1.1084
Cluster-11327.68329	PREDICTED: sn1-specific diacylglycerol lipase beta [Tribolium castaneum]>gi|270013706|gb|EFA10154.1| hypothetical protein TcasGA2_TC012342 [Tribolium castaneum]	1.191
Cluster-11327.66330	NADP-dependent malic enzyme [Zootermopsis nevadensis]	1.6794
Cluster-11327.58790	PREDICTED: neo-calmodulin-like isoform X4 [Bombus terrestris]>gi|815928242|ref|XP_012248616.1| PREDICTED: neo-calmodulin-like isoform X5 [Bombus impatiens]	1.7182
Cluster-11327.63200	hypothetical protein [Coptotermes formosanus]	6.3732
Cluster-11327.60167	PREDICTED: fucose mutarotase isoform X1 [Oryzias latipes]	1.3142
Cluster-11327.72638	PREDICTED: uncharacterized protein LOC105698441 isoform X13 [Orussus abietinus]	6.342
Cluster-11327.57300	PREDICTED: uncharacterized protein LOC103507240 [Diaphorina citri]	1.7318
Cluster-11327.60252	Echinoderm microtubule-associated protein-like 1, partial [Zootermopsis nevadensis]	1.699
Cluster-11327.59722	heat shock protein 90 [Oxya chinensis]	1.4743
Cluster-11327.56261	takeout-like [Acyrthosiphon pisum]>gi|239789998|dbj|BAH71589.1| ACYPI006265 [Acyrthosiphon pisum]	4.2805
Cluster-11327.51035	cellulase [Teleogryllus emma]>gi|167859905|gb|ACA04897.1| cellulase [Teleogryllus emma]	2.8985
Cluster-11327.55436	28S ribosomal protein S17, mitochondrial [Zootermopsis nevadensis]	1.3429
Cluster-11327.71297	3,2-trans-enoyl-CoA isomerase, mitochondrial	2.3797

	[Zootermopsis nevadensis]	
Cluster-11327.59147	PREDICTED: basement membrane-specific heparan sulfate proteoglycan core protein [Cerapachys biroi]	1.0415
Cluster-11327.48328	NADP-specific isocitrate dehydrogenase [Riptortus pedestris]	1.2401
Cluster-11327.47512	hypothetical protein L798_11789 [Zootermopsis nevadensis]	2.9068
Cluster-11327.54938	hypothetical protein L798_07794 [Zootermopsis nevadensis]	5.7848
Cluster-11327.56313	RecName: Full=Endocuticle structural glycoprotein SgAbd-3 [Schistocerca gregaria]	6.9938
Cluster-11327.47907	deviate [Reticulitermes flavipes]	8.0493
Cluster-11327.64429	PREDICTED: N-acylneuraminate-9-phosphatase [Tribolium castaneum]	4.8662
Cluster-11327.60892	hypothetical protein TcasGA2_TC014364 [Tribolium castaneum]	1.2448
Cluster-11327.62904	PREDICTED: potassium voltage-gated channel protein Shaker isoform X1 [Tribolium castaneum]	1.2231
Cluster-11327.56861	PREDICTED: putative leucine-rich repeat-containing protein DDB_G0290503 [Atta cephalotes]	1.6614
Cluster-11327.56009	PREDICTED: twitchin [Atta cephalotes]	7.9587
Cluster-11327.60204	Endoplasmic reticulum protein ERp29 [Zootermopsis nevadensis]	1.2174
Cluster-11327.60205	AGAP012048-PA [Anopheles gambiae str. PEST]	1.9214
Cluster-11327.54877	obstructor D1 [Locusta migratoria]	2.6234
Cluster-11327.59770	hypothetical protein TcasGA2_TC004196 [Tribolium castaneum]	5.6617
Cluster-11327.60623	Endocuticle structural glycoprotein SgAbd-2, partial [Zootermopsis nevadensis]	3.2448
Cluster-11327.58194	hypothetical protein L798_12962 [Zootermopsis nevadensis]	1.3681
Cluster-11327.58203	Very long-chain specific acyl-CoA dehydrogenase, mitochondrial [Zootermopsis nevadensis]	3.5971
Cluster-11327.55945	Phosphate carrier protein, mitochondrial [Zootermopsis nevadensis]	1.498
Cluster-11327.54759	Flightin [Zootermopsis nevadensis]	7.5085
Cluster-11327.52346	PREDICTED: uncharacterized protein LOC105696933 isoform X2 [Orussus abietinus]	1.3587
Cluster-11327.62050	Eukaryotic translation initiation factor 3 subunit A [Zootermopsis nevadensis]	1.4471
Cluster-11327.63443	hypoxia-inducible factor 1 alpha, partial [Locusta migratoria]	1.6751
Cluster-11327.56651	60S ribosomal protein L4 [Zootermopsis nevadensis]	5.5443
Cluster-11327.56656	PREDICTED: titin-like [Athalia rosae]	3.1881
Cluster-11327.58422	conserved hypothetical protein [Pediculus humanus corporis]	2.7416
Cluster-11327.47548	E3 ubiquitin-protein ligase KCMF1 [Zootermopsis nevadensis]	4.9251
Cluster-11327.44932	Circadian clock-controlled protein [Zootermopsis	3.0301

	nevadensis]	
Cluster-11327.64751	PREDICTED: protein takeout-like [Diaphorina citri]	1.348
Cluster-11327.60185	Pre-mRNA-splicing factor 38B [Zootermopsis nevadensis]	8.9884
Cluster-11327.61509	PREDICTED: uncharacterized protein LOC105455018 isoform X4 [Wasmannia auropunctata]	8.2915
Cluster-11327.56974	RecName: Full=Arginine kinase; Short=AK [Schistocerca americana]	3.4291
Cluster-11327.66549	PREDICTED: CD109 antigen-like isoform X2 [Athalia rosae]	1.7974
Cluster-11327.63897	Phosphorylase b kinase gamma catalytic chain, skeletal muscle isoform [Zootermopsis nevadensis]	3.0151
Cluster-11327.60159	Acyl carrier protein, mitochondrial [Zootermopsis nevadensis]	1.2847
Cluster-11327.53152	hypothetical protein L798_02839 [Zootermopsis nevadensis]	2.6066
Cluster-11327.67487	ADP-sugar pyrophosphatase-like protein [Coptotermes formosanus]	1.3955
Cluster-11327.66169	PREDICTED: ejaculatory bulb-specific protein 3-like [Fopius arisanus]	1.5214
Cluster-11327.47764	hypothetical protein L798_00836 [Zootermopsis nevadensis]	4.3129
Cluster-11327.54368	Tuberin [Zootermopsis nevadensis]	1.0269
Cluster-11327.50779	L-lactate dehydrogenase [Zootermopsis nevadensis]	1.2012
Cluster-11327.61685	obstructor C [Locusta migratoria]	1.2703
Cluster-11327.55064	D-beta-hydroxybutyrate dehydrogenase, mitochondrial [Zootermopsis nevadensis]	1.7109
Cluster-11327.58935	RING finger protein nhl-1 [Zootermopsis nevadensis]	1.668
Cluster-11327.56575	hypothetical protein L798_04004 [Zootermopsis nevadensis]	2.5936
Cluster-11327.61255	unkown protein [Riptortus pedestris]	1.5279
Cluster-11327.61251	hypothetical protein YQE_06114, partial [Dendroctonus ponderosae]	2.5811
Cluster-11327.54198	Mitochondrial import inner membrane translocase subunit TIM44 [Zootermopsis nevadensis]	1.2733
Cluster-11327.58010	hypothetical protein L798_03331, partial [Zootermopsis nevadensis]	1.1713
Cluster-11327.56106	muscle LIM protein-like protein isoform A variant 1 [Bombus hypocrita]	2.561
Cluster-11327.59297	RecName: Full=Endocuticle structural glycoprotein ABD-4 [Locusta migratoria]	2.7189
Cluster-11327.58807	hypothetical protein L798_04846 [Zootermopsis nevadensis]	5.7696
Cluster-11327.56401	PREDICTED: signal transducer and activator of transcription 5B-like [Apis dorsata]	5.5543
Cluster-11327.58674	PREDICTED: histone H2A-like [Cerapachys biroi]	5.7842
Cluster-11327.66683	PREDICTED: myc box-dependent-interacting	1.6783

	protein 1 isoform X6 [Monomorium pharaonis]	
Cluster-11327.69934	Protein-L-isoaspartate(D-aspartate) O-methyltransferase [Zootermopsis nevadensis]	5.7172
Cluster-11327.68286	Glutaredoxin 3 [Zootermopsis nevadensis]	1.462
Cluster-11327.57035	neuroparsin 3 precursor [Schistocerca gregaria]	1.5858
Cluster-11327.58279	putative phospholipid-transporting ATPase ID [Zootermopsis nevadensis]	4.5798
Cluster-11327.50441	PREDICTED: microtubule-associated protein futsch [Bombus impatiens]	4.4692
Cluster-11327.59924	PREDICTED: skin secretory protein xP2-like [Acyrthosiphon pisum]	1.5058
Cluster-11327.57515	Glutamate synthase [NADH], amyloplastic [Zootermopsis nevadensis]	8.1953
Cluster-11327.56645	PREDICTED: tyrosine 3-monooxygenase [Orussus abietinus]	2.6276
Cluster-11327.59188	PREDICTED: uncharacterized protein LOC105701830 isoform X2 [Orussus abietinus]	5.0703
Cluster-11327.59027	Serine palmitoyltransferase 2 [Zootermopsis nevadensis]	1.7969
Cluster-11327.69039	takeout-like protein 4 [Locusta migratoria]	6.4371
Cluster-11327.69031	hexamerin-like protein 1 [Locusta migratoria]	5.3233
Cluster-11327.56457	hexamerin-like protein 1 [Locusta migratoria]	4.9983
Cluster-11327.67335	hypothetical protein TcasGA2_TC013627 [Tribolium castaneum]	7.8617
Cluster-11327.57605	ATP-synthase subunit beta [Schistocerca gregaria]	5.5797
Cluster-11327.60541	RecName: Full=Endocuticle structural glycoprotein SgAbd-1 [Schistocerca gregaria]	6.0653
Cluster-11327.44291	PREDICTED: uncharacterized protein LOC105563704 isoform X2 [Vollenhovia emeryi]	5.4187
Cluster-11327.64872	PREDICTED: uncharacterized protein LOC105556100 [Vollenhovia emeryi]	3.7802
Cluster-11327.61511	hypothetical protein L798_08259, partial [Zootermopsis nevadensis]	6.3911
Cluster-11327.69746	hypothetical protein L798_08009 [Zootermopsis nevadensis]	6.5367
Cluster-11327.63630	Protein LZIC [Zootermopsis nevadensis]	5.3966
Cluster-11327.50594	Protein BAT2-like [Zootermopsis nevadensis]	7.3238
Cluster-11327.56618	Paramyosin, short form [Harpegnathos saltator]	6.851
Cluster-11327.58137	ER protein gp78 [Locusta migratoria]	7.8645
Cluster-11327.56200	PREDICTED: uncharacterized protein LOC664073 isoform X1 [Tribolium castaneum]	5.2048
Cluster-11327.64202	PREDICTED: uncharacterized protein LOC105703065 [Orussus abietinus]	6.3647
Cluster-11327.64205	hypothetical protein TcasGA2_TC004196 [Tribolium castaneum]	6.7596
Cluster-11327.69576	hypothetical protein TcasGA2_TC004196 [Tribolium castaneum]	6.5521
Cluster-11327.72347	takeout-like protein 5 [Locusta migratoria]	8.8357
Cluster-11327.67219	hypothetical protein TcasGA2_TC011099 [Tribolium castaneum]	9.1288
Cluster-11327.50938	PREDICTED: zinc finger BED domain-containing protein 5-like [Microplitis demolitor]	6.8194

Cluster-11327.59577	Sorbitol dehydrogenase [Zootermopsis nevadensis]	6.1134
Cluster-11327.62710	hypothetical protein Phum_PHUM283590 [Pediculus humanus corporis	9.7385
Cluster-11327.50244	nuclear valosin-containing [Lasius niger]	2.4975
Cluster-11327.62863	Brain protein, putative [Pediculus humanus corporis]	7.9736
Cluster-11327.56790	cytochrome b [Oedaleus decorus asiaticus]	7.2293
Cluster-11327.52765	TIMELESS [Gryllus bimaculatus]	7.2528
Cluster-11327.60278	Fumarate hydratase, mitochondrial [Zootermopsis nevadensis]	7.8454
Cluster-11327.59705	hypothetical protein L798_02578 [Zootermopsis nevadensis]	5.3105
Cluster-11327.59096	GAPDH [Locusta migratoria]	5.6199
Cluster-11327.66942	Carnitine O-palmitoyltransferase 2, mitochondrial [Zootermopsis nevadensis]	3.4232
Cluster-11327.84352	glutathione S-transferase domain-containing protein [Polysphondylium pallidum PN500]	6.7596
Cluster-11327.85381	theta glutathione S-transferase [Locusta migratoria]	6.5521
Cluster-11327.80231	PREDICTED: probable cytochrome P450 6a14 [Acyrthosiphon pisum]	8.8357
Cluster-21310.0	PREDICTED: cytochrome P450 4V2-like [Bos mutus] P450 4V2 [Bos mutus]	9.1288
Cluster-11327.70406	cytochrome P450 9E1 [Diploptera punctata]	6.8194
Cluster-12457.0	cytochrome P450 monooxygenase, putative [Acanthamoeba castellanii str. Neff]	6.1134
Cluster-11327.66426	carboxylesterase [Oxya chinensis]	9.7385
Cluster-11327.71822	carboxylesterase [Oxya chinensis]	2.4975
Cluster-11327.34656	cytochrome P450 9E1 [Diploptera punctata]	7.9736
Cluster-11327.56437	PREDICTED: cytochrome P450 4g15 [Orussus abietinus]	7.2293
Cluster-11327.36337	carboxylesterase [Oxya chinensis]	8.03
Cluster-11327.73547	PREDICTED: cytochrome P450 6a2 [Tribolium castaneum]	8.4514
Cluster-11327.6823	putative cytochrome P450 6a14 [Zootermopsis nevadensis]	7.2528
Cluster-11327.50506	glutathione S-transferase sigma 4 [Locusta migratoria]	7.8454
Cluster-11327.59598	carboxylesterase [Locusta migratoria]	5.3105
Cluster-11327.100541	PREDICTED: cytochrome P450 6j1-like [Acyrthosiphon pisum]	5.6199
Cluster-4727.0	PREDICTED: probable cytochrome P450 4aa1 [Nasonia vitripennis]	3.4232
Cluster-11327.79488	PREDICTED: probable cytochrome P450 6a23 [Tribolium castaneum]	4.6943
Cluster-11327.78130	carboxylesterase [Oxya chinensis]	4.9269
Cluster-11327.55763	superoxide dismutase [Schistocerca gregaria]	7.6322
Cluster-11327.49164	delta glutathione S-transferase [Locusta migratoria]	9.2654
Cluster-11327.22262	UDP-glucuronosyltransferase 2C1 [Zootermopsis nevadensis]	3.6205

Cluster-11327.56874	Peroxidase [Zootermopsis nevadensis]	8.0493
Cluster-24423.0	copper/zinc superoxide dismutase [Mauremys reevesii]	4.8662
Cluster-11327.25393	UDP-glucuronosyltransferase 2C1 [Zootermopsis nevadensis]	1.2448
Cluster-11327.59722	heat shock protein 90 [Oxya chinensis]	1.2231
Cluster-11327.87593	beta-glucosidase, partial [Coptotermes formosanus]	2.4429
Cluster-11327.3892	beta-glucosidase [Periplaneta americana]	1.6614
Cluster-11327.46783	Lactase-phlorizin hydrolase [Zootermopsis nevadensis]	7.9587
Cluster-11327.37866	UDP-glucuronosyltransferase 2C1 [Zootermopsis nevadensis]	1.2174
Cluster-11327.54468	Peroxidase [Zootermopsis nevadensis]	1.9214
Cluster-11327.47272	PREDICTED: peroxidase [Tribolium castaneum]	1.0595
Cluster-11327.4404	catalase isozyme 2 [Capsaspora owczarzaki ATCC 30864]	5.1419
Cluster-11327.58858	Lactase-phlorizin hydrolase [Zootermopsis nevadensis]	2.6234
Cluster-11327.46674	pelle [Litopenaeus vannamei]	5.6617
Cluster-11327.54592	Lactase-phlorizin hydrolase [Zootermopsis nevadensis]	4.1197
Cluster-11327.84060	beta-glucosidase [Periplaneta americana]	1.8551
Cluster-11327.48874	catalase, partial [Schistocerca gregaria]	3.2448
Cluster-11327.33691	UDP-glucuronosyltransferase 2B31 [Zootermopsis nevadensis]	2.9272
Cluster-11327.24983	UDP-glucuronosyltransferase 2B1 [Zootermopsis nevadensis]	8.9884
Cluster-11327.93898	cytochrome P450 9E1 [Diploptera punctata]	8.2915
Cluster-11327.76409	catalase isozyme 2 [Capsaspora owczarzaki ATCC 30864]	3.4291
Cluster-11327.54882	cytochrome P450 6 K1[Locusta migratoria manilensis]	6.3763
Cluster-11327.75652	FoxO protein [Blattella germanica]	8.3962
Cluster-11327.57156	heat shock protein 19.8 [Oxya chinensis]	4.329

Table S6 The down-regulated genes (adjusted *P* values < 0.05, $|\log_2$.Fold_change$| > 1$) of *O. asiaticus* feeding rutin-treated foods compared with individuals feeding no rutin-treated foods.

Gene ID	Description	Fold change
Cluster-11327.66427	Na+/K+-ATPase alpha2 [Locusta migratoria]	-2.4535
Cluster-11327.54502	NADH dehydrogenase [ubiquinone] 1 alpha subcomplex subunit 10, mitochondrial [Zootermopsis nevadensis]	-2.2525
Cluster-11327.52235	PREDICTED: acetyl-CoA acetyltransferase, mitochondrial isoform X3 [Monomorium pharaonis]	-1.7426
Cluster-11327.56806	PREDICTED: alpha-1,6-mannosyl-glycoprotein 2-beta-N-acetylglucosaminyltransferase isoform	-1.4561

	X2 [Tribolium castaneum]	
Cluster-11327.60488	endoplasmin [Locusta migratoria]	-1.1646
Cluster-11327.53295	Tyrosine-protein kinase Src42A [Zootermopsis nevadensis]	-3.1291
Cluster-11327.63162	PREDICTED: fatty acid synthase-like [Microplitis demolitor]	-1.5154
Cluster-11327.56187	Lipase 3 [Zootermopsis nevadensis]	-1.1585
Cluster-11327.82672	RUN and FYVE domain-containing protein 2 [Zootermopsis nevadensis]	-1.3401
Cluster-11327.11957	PREDICTED: cytochrome P450 26A1 [Larimichthys crocea]	-1.1059
Cluster-11327.86192	hypothetical protein X777_09218 [Cerapachys biroi]	-1.4478
Cluster-11327.57724	PREDICTED: very long-chain-fatty-acid--CoA ligase bubblegum isoform X2 [Tribolium castaneum]	-5.9576
Cluster-11327.49351	Transcription initiation factor TFIID subunit 2 [Zootermopsis nevadensis]	-1.5925
Cluster-11327.60249	troponin C [Papilio xuthus]	-2.1733
Cluster-11327.100352	unnamed protein product [Vitrella brassicaformis CCMP3155]	-1.2417
Cluster-11327.55587	PREDICTED: glycogen-binding subunit 76A [Tribolium castaneum]	-2.0698
Cluster-11327.54616	Fumarate hydratase, mitochondrial [Zootermopsis nevadensis]	-1.049
Cluster-11327.51896	phosphoenolpyruvate carboxykinase [Blattella germanica]	-1.595
Cluster-11327.58073	Carnitine O-palmitoyltransferase 2, mitochondrial [Zootermopsis nevadensis]	-1.3668
Cluster-11327.60788	hypothetical protein L798_03479 [Zootermopsis nevadensis]	-2.4453
Cluster-11327.57904	PREDICTED: fatty acid synthase [Acyrthosiphon pisum]	-3.0473
Cluster-11327.46292	PREDICTED: putative carbonic anhydrase 3 [Plutella xylostella]	-5.8987
Cluster-11327.57771	Hexokinase type 2 [Zootermopsis nevadensis]	-1.0889
Cluster-11327.24787	hypothetical protein L798_01066, partial [Zootermopsis nevadensis]	-5.8758
Cluster-11327.75104	PREDICTED: uncharacterized protein KIAA1109 isoform X8 [Camponotus floridanus]	-1.0387
Cluster-11327.27064	hypothetical protein NEMVEDRAFT_v1g231585 [Nematostella vectensis]	-1.6175
Cluster-8319.0	PREDICTED: NADH-quinone oxidoreductase	-2.4453

	subunit C/D-like, partial [Macaca mulatta]	
Cluster-11327.45680	6.3 kDa salivary peptide [Anopheles darlingi]	-2.0762
Cluster-11327.61084	Protein held out wings [Zootermopsis nevadensis]	-1.0639
Cluster-11327.57165	ATP synthase lipid-binding protein, mitochondrial [Zootermopsis nevadensis]	-2.0724
Cluster-11327.55927	calreticulin [Schistocerca gregaria]	-3.4873
Cluster-11327.59611	glutamine synthetase [Schistocerca gregaria]	-3.0374
Cluster-11327.66457	PREDICTED: GDP-D-glucose phosphorylase 1-like isoform X2 [Linepithema humile]	-2.5915
Cluster-11327.63200	hypothetical protein [Coptotermes formosanus]	-1.7206
Cluster-11327.53657	RAC serine/threonine-protein kinase [Zootermopsis nevadensis]	-6.3537
Cluster-11327.67468	hypothetical protein L798_06748 [Zootermopsis nevadensis]	-1.7284
Cluster-11327.71297	3,2-trans-enoyl-CoA isomerase, mitochondrial [Zootermopsis nevadensis]	-1.4607
Cluster-11327.29145	hypothetical protein L798_02293 [Zootermopsis nevadensis]	-1.4262
Cluster-11327.39669	Carnitine O-palmitoyltransferase 2, mitochondrial [Zootermopsis nevadensis]	-2.1807
Cluster-11327.44964	Lipase 3 [Zootermopsis nevadensis]	-1.6723
Cluster-11327.64830	Proto-oncogene tyrosine-protein kinase ROS [Zootermopsis nevadensis]	-1.3556
Cluster-11327.60204	Endoplasmic reticulum protein ERp29 [Zootermopsis nevadensis]	-5.2737
Cluster-11327.70970	RAC serine/threonine-protein kinase [Zootermopsis nevadensis]	-2.7342
Cluster-11327.53292	neuroparsin 3 precursor [Schistocerca gregaria]	-1.1926
Cluster-11327.59047	Hexokinase type 2 [Zootermopsis nevadensis]	-1.28
Cluster-11327.62190	PREDICTED: paxillin-like isoform X4 [Athalia rosae]	-3.8426
Cluster-11327.45348	PREDICTED: F-box only protein 11 [Apis florea]	-1.101
Cluster-11327.17732	hypothetical protein SAMD00019534_095720, partial [Acytostelium subglobosum LB1]	-2.7128
Cluster-14403.0	hypothetical protein L798_07136 [Zootermopsis nevadensis]	-1.4249
Cluster-11327.63434	PREDICTED: paxillin-like isoform X3 [Athalia rosae]	-3.0155
Cluster-11327.55472	Collagen alpha-2(I) chain, partial [Zootermopsis nevadensis]	-1.4389
Cluster-11327.54970	Hydroxyacyl-coenzyme A dehydrogenase, mitochondrial [Zootermopsis nevadensis]	-2.2
Cluster-11327.57605	ATP-synthase subunit beta [Schistocerca gregaria]	-1.7978

Cluster-11327.64046	Short/branched chain specific acyl-CoA dehydrogenase, mitochondrial [Zootermopsis nevadensis]	-8.0497
Cluster-11327.10917	PREDICTED: alcohol dehydrogenase class-3-like [Astyanax mexicanus]	-1.0128
Cluster-11327.56167	NADH dehydrogenase subunit 5 [Oedaleus decorus asiaticus]	-2.887
Cluster-11327.50593	PREDICTED: troponin C [Ceratosolen solmsi marchali]	-2.4958
Cluster-11327.50592	PREDICTED: troponin C [Ceratosolen solmsi marchali]	-1.883
Cluster-11327.57481	alpha-amylase [Blattella germanica]	-2.1461
Cluster-11327.58763	ATP synthase lipid-binding protein, mitochondrial [Zootermopsis nevadensis]	-1.0159
Cluster-11327.57866	ATP-synthase subunit beta [Schistocerca gregaria]	-1.2015
Cluster-11327.64619	PREDICTED: paxillin-like isoform X7 [Athalia rosae]	-1.2187
Cluster-11327.95868	hypothetical protein L798_03265 [Zootermopsis nevadensis]	-6.1502
Cluster-11327.55712	ER protein gp78 [Locusta migratoria]	-2.1685
Cluster-11327.89549	Histone-lysine N-methyltransferase PRDM9 [Crassostrea gigas]	-2.9731
Cluster-11327.48357	6.3 kDa salivary peptide [Anopheles darlingi]	-1.296
Cluster-11327.57428	PREDICTED: putative carbonic anhydrase 3 [Plutella xylostella]	-1.1916
Cluster-11327.57882	allergen Bla g 6.0101 [Blattella germanica]	-2.3788
Cluster-11327.16428	PREDICTED: ATP synthase subunit beta, mitochondrial-like, partial [Pelodiscus sinensis]	-1.4467
Cluster-11327.60586	PREDICTED: glycogen-binding subunit 76A [Tribolium castaneum]	-1.3519
Cluster-11327.90628	PREDICTED: GDP-D-glucose phosphorylase 1-like isoform X2 [Linepithema humile]	-4.4473
Cluster-11327.51895	phosphoenolpyruvate carboxykinase [Blattella germanica]	-1.0619
Cluster-11327.57198	Very long-chain specific acyl-CoA dehydrogenase, mitochondrial [Zootermopsis nevadensis]	-2.865
Cluster-11327.56790	cytochrome b [Oedaleus decorus asiaticus]	-1.8328
Cluster-11327.56795	protein disulfide-isomerase [Schistocerca gregaria]	-1.4962
Cluster-11327.33845	PREDICTED: citrate lyase subunit beta-like protein, mitochondrial [Callorhinchus milii]	-1.1988
Cluster-11327.10733	PREDICTED: pectinesterase QRT1-like	-3.1192

	[Pantholops hodgsonii]	
Cluster-11327.66942	Carnitine O-palmitoyltransferase 2, mitochondrial [Zootermopsis nevadensis]	-1.9621
Cluster-11327.53067	Tenascin-X [Zootermopsis nevadensis]	-1.5434
Cluster-11327.63689	Microsomal triglyceride transfer protein large subunit [Zootermopsis nevadensis]	-4.3019
Cluster-11327.59161	PREDICTED: carnitine O-acetyltransferase-like, partial [Plutella xylostella]	-1.386
Cluster-11327.55961	Translation initiation factor IF-2 [Cricetulus griseus]	-1.9506
Cluster-11327.56438	Protein hu-li tai shao [Zootermopsis nevadensis]	-1.259
Cluster-11327.46754	PREDICTED: aminopeptidase N [Pogonomyrmex barbatus]	-6.361
Cluster-11327.57432	PREDICTED: uncharacterized protein LOC105360525 [Ceratosolen solmsi marchali]	-1.6199
Cluster-11327.56020	PREDICTED: flexible cuticle protein 12 [Tribolium castaneum]	-1.5987
Cluster-11327.56022	Electron transfer flavoprotein subunit beta [Zootermopsis nevadensis]	-1.4175
Cluster-11327.48398	PREDICTED: dynamin-like 120 kDa protein, mitochondrial isoform X1 [Athalia rosae] rosae]	-1.1528
Cluster-11327.58041	C3 and PZP-like alpha-2-macroglobulin domain-containing protein 8 [Zootermopsis nevadensis]	-3.0264
Cluster-11327.53166	hypothetical protein L798_07481 [Zootermopsis nevadensis]	-1.8986
Cluster-11327.56485	PREDICTED: muscle M-line assembly protein unc-89 [Athalia rosae]	-4.9828
Cluster-11327.52447	chemosensory protein [Chilo auricilius]	-2.9454
Cluster-11327.57341	hypothetical protein L798_15728, partial [Zootermopsis nevadensis]	-6.7963
Cluster-11327.57178	LIM domain and actin-binding protein 1 [Zootermopsis nevadensis]	-2.1958
Cluster-11327.56226	Cytochrome b-c1 complex subunit 2, mitochondrial [Zootermopsis nevadensis]	-2.0107
Cluster-11327.51794	PREDICTED: oxidation resistance protein 1 isoform X7 [Monomorium pharaonis]	-4.2029
Cluster-11327.62704	PREDICTED: acylphosphatase-1-like [Apis florea]	-1.9031
Cluster-11327.48215	Collagen alpha-1(XI) chain, partial [Zootermopsis nevadensis]	-1.2371
Cluster-11327.53136	PREDICTED: delta-1-pyrroline-5-carboxylate dehydrogenase, mitochondrial isoform X2	-3.9713

	[Acyrthosiphon pisum]	
Cluster-11327.56041	PREDICTED: muscle-specific protein 20-like [Megachile rotundata]	-4.206
Cluster-11327.56048	40S ribosomal protein S5a [Zootermopsis nevadensis]	-1.0025
Cluster-11327.53645	arylphorin hexamerin-like protein 2 [Romalea microptera]	-1.5839
Cluster-11327.51481	RecName: Full=Rhodopsin; Short=Opsin [Sphodromantis sp.]	-1.2065
Cluster-11327.67900	Mite allergen Lep d 7 [Zootermopsis nevadensis]	-1.6277
Cluster-11327.55944	PREDICTED: titin isoform X3 [Bombus terrestris]	-1.6142
Cluster-11327.46428	PREDICTED: protein Malvolio isoform X2 [Microplitis demolitor]	-1.7885
Cluster-11327.63404	60s ribosomal protein l7 [Lasius niger]	-1.4073
Cluster-11327.58073	Carnitine O-palmitoyltransferase 2, mitochondrial [Zootermopsis nevadensis]	-2.6972
Cluster-11327.55584	60S ribosomal protein L12 [Zootermopsis nevadensis]	-2.4584
Cluster-11327.56690	Aspartate aminotransferase, mitochondrial [Zootermopsis nevadensis]	-2.014
Cluster-11327.61337	hypothetical protein KGM_12267 [Danaus plexippus]	-1.6442
Cluster-11327.60785	uncharacterized protein [Coptotermes formosanus]	-1.9223
Cluster-11327.50627	hypothetical protein YQE_04947, partial [Dendroctonus ponderosae]	-1.9981
Cluster-11327.57908	laminin A chain, putative [Pediculus humanus corporis]	-1.3069
Cluster-11327.54515	Ligand of Numb protein X 2 [Zootermopsis nevadensis]	-1.0191
Cluster-11327.56584	PREDICTED: malate dehydrogenase, cytoplasmic [Musca domestica]	-3.7314
Cluster-11327.72662	endonuclease-reverse transcriptase [Eyprepocnemis plorans plorans]	-3.2045
Cluster-11327.52547	PREDICTED: pyridoxal kinase [Tribolium castaneum] [Tribolium castaneum]	-1.3821
Cluster-11327.48958	PREDICTED: ras and Rab interactor 1 [Danio rerio]	-5.7719
Cluster-11327.51972	hypothetical protein L798_04814 [Zootermopsis nevadensis]	-2.8647
Cluster-11327.59293	RecName: Full=Endocuticle structural glycoprotein ABD-4 [Locusta migratoria]	-1.7923

Cluster-11327.57470	Dehydrogenase/reductase SDR family member 11 [Zootermopsis nevadensis]	-3.5422
Cluster-11327.70020	hypothetical protein TcasGA2_TC006727 [Tribolium castaneum]	-1.7069
Cluster-11327.61361	PREDICTED: uncharacterized protein LOC105556100 [Vollenhovia emeryi]	-1.2856
Cluster-11327.61362	PREDICTED: uncharacterized protein LOC105556100 [Vollenhovia emeryi]	-7.8157
Cluster-11327.62606	putative adenylate kinase isoenzyme F38B2.4 [Zootermopsis nevadensis]	-6.7951
Cluster-11327.49642	enzymatic polyprotein, putative [Pediculus humanus corporis]	-1.8571
Cluster-11327.57957	Endocuticle structural glycoprotein SgAbd-2, partial [Zootermopsis nevadensis]	-3.7556
Cluster-11327.59905	hypothetical protein L798_14756 [Zootermopsis nevadensis]	-2.2209
Cluster-11327.58542	actin-depolymerizing factor 1 [Coptotermes formosanus]	-4.9504
Cluster-11327.60010	PREDICTED: PDZ and LIM domain protein Zasp-like isoform X3 [Megachile rotundata]	-1.1265
Cluster-11327.61768	PREDICTED: NAD kinase-like isoform X10 [Diaphorina citri]	-1.001
Cluster-11327.52948	obstructor A1 [Locusta migratoria]	-1.0644
Cluster-11327.67601	vermiform [Anopheles darlingi]	-8.2397
Cluster-11327.62388	PREDICTED: muscle M-line assembly protein unc-89 isoform X2 [Monomorium pharaonis]	-1.021
Cluster-11327.68329	PREDICTED: sn1-specific diacylglycerol lipase beta [Tribolium castaneum]	-4.2706
Cluster-11327.66330	NADP-dependent malic enzyme [Zootermopsis nevadensis]	-1.6399
Cluster-11327.52281	Clathrin light chain [Zootermopsis nevadensis]	-1.8353
Cluster-11327.58790	PREDICTED: neo-calmodulin-like isoform X4 [Bombus terrestris]	-8.0161
Cluster-11327.63200	hypothetical protein [Coptotermes formosanus]	-4.9655
Cluster-11327.60167	PREDICTED: fucose mutarotase isoform X1 [Oryzias latipes]	-1.9452
Cluster-11327.72638	PREDICTED: uncharacterized protein LOC105698441 isoform X13 [Orussus abietinus]	-1.8867
Cluster-11327.57300	PREDICTED: uncharacterized protein LOC103507240 [Diaphorina citri]	-2.2552
Cluster-11327.60252	Echinoderm microtubule-associated protein-like 1, partial [Zootermopsis nevadensis]	-1.9014
Cluster-11327.55482	Transposable element Tcb1 transposase, partial	-1.2893

	[Stegodyphus mimosarum]	
Cluster-11327.56261	takeout-like [Acyrthosiphon pisum]	-1.6723
Cluster-11327.51035	cellulase [Teleogryllus emma]	-1.6028
Cluster-11327.61261	conserved hypothetical protein [Pediculus humanus corporis]	-2.3664
Cluster-11327.55436	28S ribosomal protein S17, mitochondrial [Zootermopsis nevadensis]	-1.1134
Cluster-11327.71297	3,2-trans-enoyl-CoA isomerase, mitochondrial [Zootermopsis nevadensis]	-3.2304
Cluster-11327.67654	Endocuticle structural glycoprotein SgAbd-9, partial [Zootermopsis nevadensis]	-6.9678
Cluster-11327.59147	PREDICTED: basement membrane-specific heparan sulfate proteoglycan core protein [Cerapachys biroi]	-1.4277
Cluster-11327.48328	NADP-specific isocitrate dehydrogenase [Riptortus pedestris]	-4.4288
Cluster-11327.47512	hypothetical protein L798_11789 [Zootermopsis nevadensis]	-5.7805
Cluster-11327.54938	hypothetical protein L798_07794 [Zootermopsis nevadensis]	-1.5919
Cluster-11327.56313	RecName: Full=Endocuticle structural glycoprotein SgAbd-3 [Schistocerca gregaria]	-1.242
Cluster-11327.47907	deviate [Reticulitermes flavipes]	-3.6988
Cluster-11327.64429	PREDICTED: N-acylneuraminate-9-phosphatase [Tribolium castaneum]	-5.9396
Cluster-11327.60892	hypothetical protein TcasGA2_TC014364 [Tribolium castaneum]	-1.6544
Cluster-11327.62904	PREDICTED: potassium voltage-gated channel protein Shaker isoform X1 [Tribolium castaneum]	-4.4836
Cluster-11327.56861	PREDICTED: putative leucine-rich repeat-containing protein DDB_G0290503 [Atta cephalotes]	-2.245
Cluster-11327.56009	PREDICTED: twitchin [Atta cephalotes]	-5.5962
Cluster-11327.60204	Endoplasmic reticulum protein ERp29 [Zootermopsis nevadensis]	-6.0984
Cluster-11327.60205	AGAP012048-PA [Anopheles gambiae str. PEST]	-4.6091
Cluster-11327.54877	obstructor D1 [Locusta migratoria]	-2.1551
Cluster-11327.59770	hypothetical protein TcasGA2_TC004196 [Tribolium castaneum]	-1.4461
Cluster-11327.60623	Endocuticle structural glycoprotein SgAbd-2, partial [Zootermopsis nevadensis]	-3.7361
Cluster-11327.58194	hypothetical protein L798_12962 [Zootermopsis nevadensis]	-1.6819

Cluster-11327.58203	Very long-chain specific acyl-CoA dehydrogenase, mitochondrial [Zootermopsis nevadensis]	-5.0314
Cluster-11327.55945	Phosphate carrier protein, mitochondrial [Zootermopsis nevadensis]	-4.3801
Cluster-11327.54759	Flightin [Zootermopsis nevadensis]	-2.2911
Cluster-11327.52346	PREDICTED: uncharacterized protein LOC105696933 isoform X2 [Orussus abietinus]	-3.1176
Cluster-11327.62050	Eukaryotic translation initiation factor 3 subunit A [Zootermopsis nevadensis]	-1.5759
Cluster-11327.63443	hypoxia-inducible factor 1 alpha, partial [Locusta migratoria]	-5.78
Cluster-11327.56651	60S ribosomal protein L4 [Zootermopsis nevadensis]	-1.7219
Cluster-11327.56656	PREDICTED: titin-like [Athalia rosae]	-3.5228
Cluster-11327.49673	PREDICTED: uncharacterized protein LOC100573494 [Acyrthosiphon pisum]	-1.4902
Cluster-11327.54333	Neuroblastoma suppressor of tumorigenicity 1 [Camponotus floridanus]	-6.4672
Cluster-11327.58422	conserved hypothetical protein [Pediculus humanus corporis]	-1.6478
Cluster-11327.47548	E3 ubiquitin-protein ligase KCMF1 [Zootermopsis nevadensis]	-1.2253
Cluster-11327.44932	Circadian clock-controlled protein [Zootermopsis nevadensis]	-2.4685
Cluster-11327.64751	PREDICTED: protein takeout-like [Diaphorina citri]	-3.0486
Cluster-11327.60185	Pre-mRNA-splicing factor 38B [Zootermopsis nevadensis]	-6.6427
Cluster-11327.61509	PREDICTED: uncharacterized protein LOC105455018 isoform X4 [Wasmannia auropunctata]	-2.2703
Cluster-11327.56974	RecName: Full=Arginine kinase; Short=AK [Schistocerca americana]	-5.2141
Cluster-11327.66549	PREDICTED: CD109 antigen-like isoform X2 [Athalia rosae]	-5.1062
Cluster-11327.63897	Phosphorylase b kinase gamma catalytic chain, skeletal muscle isoform [Zootermopsis nevadensis]	-5.4903
Cluster-11327.60159	Acyl carrier protein, mitochondrial [Zootermopsis nevadensis]	-3.1289
Cluster-11327.53152	hypothetical protein L798_02839 [Zootermopsis nevadensis]	-4.8046
Cluster-11327.67487	ADP-sugar pyrophosphatase-like protein	-3.7162

	[Coptotermes formosanus]	
Cluster-11327.66169	PREDICTED: ejaculatory bulb-specific protein 3-like [Fopius arisanus]	-6.5567
Cluster-11327.57371	PREDICTED: uncharacterized protein LOC105336559 [Crassostrea gigas]	-2.886
Cluster-11327.47764	hypothetical protein L798_00836 [Zootermopsis nevadensis]	-5.1339
Cluster-11327.54368	Tuberin [Zootermopsis nevadensis]	-5.4199
Cluster-11327.56218	unknown secreted protein, partial [Riptortus pedestris]	-1.3362
Cluster-11327.50779	L-lactate dehydrogenase [Zootermopsis nevadensis]	-5.4191
Cluster-11327.61685	obstructor C [Locusta migratoria]	-5.353
Cluster-11327.54583	60S ribosomal protein L12 [Zootermopsis nevadensis]	-3.4704
Cluster-11327.55064	D-beta-hydroxybutyrate dehydrogenase, mitochondrial [Zootermopsis nevadensis]	-2.7478
Cluster-11327.58935	RING finger protein nhl-1 [Zootermopsis nevadensis]	-4.9946
Cluster-11327.55840	mitochondrial F1-ATP synthase alpha subunit [Locusta migratoria manilensis]	-3.6721
Cluster-11327.56575	hypothetical protein L798_04004 [Zootermopsis nevadensis]	-2.413
Cluster-11327.61255	unkown protein [Riptortus pedestris]	-4.9944
Cluster-11327.61251	hypothetical protein YQE_06114, partial [Dendroctonus ponderosae]	-6.5748
Cluster-11327.54198	Mitochondrial import inner membrane translocase subunit TIM44 [Zootermopsis nevadensis]	-5.8552
Cluster-11327.58010	hypothetical protein L798_03331, partial [Zootermopsis nevadensis]	-5.2152
Cluster-11327.56106	muscle LIM protein-like protein isoform A variant 1 [Bombus hypocrita]	-3.8871
Cluster-11327.59297	RecName: Full=Endocuticle structural glycoprotein ABD-4 [Locusta migratoria]	-6.4708
Cluster-11327.52195	Microsomal triglyceride transfer protein large subunit [Zootermopsis nevadensis]	-5.1038
Cluster-11327.58807	hypothetical protein L798_04846 [Zootermopsis nevadensis]	-1.9465
Cluster-11327.56401	PREDICTED: signal transducer and activator of transcription 5B-like [Apis dorsata]	-5.3733
Cluster-11327.58674	PREDICTED: histone H2A-like [Cerapachys biroi]	-5.0626
Cluster-11327.66683	PREDICTED: myc box-dependent-interacting	-4.918

	protein 1 isoform X6 [Monomorium pharaonis]	
Cluster-11327.69934	Protein-L-isoaspartate(D-aspartate) O-methyltransferase [Zootermopsis nevadensis]	-3.3065
Cluster-11327.68286	Glutaredoxin 3 [Zootermopsis nevadensis]	-5.6898
Cluster-11327.57035	neuroparsin 3 precursor [Schistocerca gregaria]	-5.6512
Cluster-11327.56343	PREDICTED: trifunctional enzyme subunit alpha, mitochondrial [Tribolium castaneum]	-3.7383
Cluster-11327.58279	putative phospholipid-transporting ATPase ID [Zootermopsis nevadensis]	-3.3993
Cluster-11327.50441	PREDICTED: microtubule-associated protein futsch [Bombus impatiens]	-1.96
Cluster-11327.59924	PREDICTED: skin secretory protein xP2-like [Acyrthosiphon pisum]	-5.1597
Cluster-11327.59817	hypothetical protein L798_04266 [Zootermopsis nevadensis]	-5.7887
Cluster-11327.54762	PREDICTED: calcium release-activated calcium channel protein 1-like isoform X2 [Orussus abietinus]	-5.4865
Cluster-11327.57515	Glutamate synthase [NADH], amyloplastic [Zootermopsis nevadensis]	-3.1331
Cluster-11327.56645	PREDICTED: tyrosine 3-monooxygenase [Orussus abietinus]	-4.21
Cluster-11327.59188	PREDICTED: uncharacterized protein LOC105701830 isoform X2 [Orussus abietinus]	-2.47
Cluster-11327.59027	Serine palmitoyltransferase 2 [Zootermopsis nevadensis]	-6.0214
Cluster-11327.54970	Hydroxyacyl-coenzyme A dehydrogenase, mitochondrial [Zootermopsis nevadensis]	-1.3824
Cluster-11327.69039	takeout-like protein 4 [Locusta migratoria]	-5.8834
Cluster-11327.67335	hypothetical protein TcasGA2_TC013627 [Tribolium castaneum]	-7.0533
Cluster-11327.57605	ATP-synthase subunit beta [Schistocerca gregaria]	-5.1894
Cluster-11327.60541	RecName: Full=Endocuticle structural glycoprotein SgAbd-1 [Schistocerca gregaria]	-2.1069
Cluster-11327.61511	hypothetical protein L798_08259, partial [Zootermopsis nevadensis]	-1.1446
Cluster-11327.69746	hypothetical protein L798_08009 [Zootermopsis nevadensis]	-6.2986
Cluster-11327.63630	Protein LZIC [Zootermopsis nevadensis]	-7.0306
Cluster-11327.55240	hypothetical protein L798_07871 [Zootermopsis nevadensis]	-2.1248
Cluster-11327.50594	Protein BAT2-like [Zootermopsis nevadensis]	-5.8987
Cluster-11327.56618	Paramyosin, short form [Harpegnathos saltator]	-5.1824

Cluster-11327.58137	ER protein gp78 [Locusta migratoria]	-1.1086
Cluster-11327.56200	PREDICTED: uncharacterized protein LOC664073 isoform X1 [Tribolium castaneum]	-1.3283
Cluster-11327.64202	PREDICTED: uncharacterized protein LOC105703065 [Orussus abietinus]	-1.1971
Cluster-11327.64205	hypothetical protein TcasGA2_TC004196 [Tribolium castaneum]	-1.5004
Cluster-11327.69576	hypothetical protein TcasGA2_TC004196 [Tribolium castaneum]	-3.1195
Cluster-11327.72347	takeout-like protein 5 [Locusta migratoria]	-2.1829
Cluster-11327.58556	putative citrate synthase 2, mitochondrial [Zootermopsis nevadensis]	-1.1192
Cluster-11327.58763	ATP synthase lipid-binding protein, mitochondrial [Zootermopsis nevadensis]	-7.1463
Cluster-11327.57866	ATP-synthase subunit beta [Schistocerca gregaria]	-1.6521
Cluster-11327.51158	hypothetical protein L798_02749 [Zootermopsis nevadensis]	-4.8653
Cluster-11327.64619	PREDICTED: paxillin-like isoform X7 [Athalia rosae]	-1.2064
Cluster-11327.54599	PREDICTED: troponin I isoform X15 [Bombus terrestris]	-2.8509
Cluster-11327.66284	Succinyl-CoA ligase [GDP-forming] subunit alpha, mitochondrial [Zootermopsis nevadensis]	-1.9403
Cluster-11327.60414	putative chitinase 3 [Zootermopsis nevadensis]	-1.3787
Cluster-11327.58450	hexamerin-like protein 1 [Locusta migratoria]	-1.3322
Cluster-11327.60975	PREDICTED: uncharacterized protein LOC105336559 [Crassostrea gigas]	-3.5023
Cluster-11327.62615	PREDICTED: glycine N-methyltransferase [Tribolium castaneum]	-1.1658
Cluster-11327.53094	Eukaryotic peptide chain release factor GTP-binding subunit ERF3B [Zootermopsis nevadensis]	-2.6315
Cluster-11327.57005	sn-glycerol-3-phosphate dehydrogenase isoform 4 [Locusta migratoria]	-1.8857
Cluster-11327.58268	chitin deacetylase 1 [Oxya chinensis]	-1.9212
Cluster-11327.66236	PREDICTED: 6-phosphogluconate dehydrogenase, decarboxylating isoform X1 [Strongylocentrotus purpuratus]	-1.5265
Cluster-11327.62635	RecName: Full=Endocuticle structural glycoprotein SgAbd-5 [Schistocerca gregaria]	-7.0814
Cluster-11327.66649	PREDICTED: uncharacterized protein LOC105556100 [Vollenhovia emeryi]	-2.0152
Cluster-11327.66641	PREDICTED: extensin-like [Diaphorina citri]	-2.009

Cluster-11327.67219	hypothetical protein TcasGA2_TC011099 [Tribolium castaneum]	-1.1438
Cluster-11327.50938	PREDICTED: zinc finger BED domain-containing protein 5-like [Microplitis demolitor]	-3.0333
Cluster-11327.72323	PREDICTED: LOW QUALITY PROTEIN: methyltransferase-like protein 5 [Microplitis demolitor]	-1.5587
Cluster-11327.55152	GJ17901 [Drosophila virilis]>gi|194141456|gb|EDW57875.1| GJ17901 [Drosophila virilis]	-1.7892
Cluster-11327.55976	mitochondrial cytochrome c [Locusta migratoria]	-1.0303
Cluster-11327.58239	Mannose-1-phosphate guanyltransferase beta [Zootermopsis nevadensis]	-3.1404
Cluster-11327.55731	delta-9 desaturase 1 [Acheta domesticus]	-2.1563
Cluster-11327.59857	PREDICTED: chromodomain-helicase-DNA-binding protein 7 isoform X5 [Wasmannia auropunctata]	-1.7269
Cluster-11327.58376	hypothetical protein RF55_16186, partial [Lasius niger]	-4.9662
Cluster-11327.58602	discs large 1 [Gryllus bimaculatus]	-2.6252
Cluster-11327.49585	Transcription factor castor [Zootermopsis nevadensis]	-1.8459
Cluster-11327.56600	enzymatic polyprotein, putative [Pediculus humanus corporis]	-3.4488
Cluster-11327.60278	Fumarate hydratase, mitochondrial [Zootermopsis nevadensis]	-1.2522
Cluster-11327.59705	hypothetical protein L798_02578 [Zootermopsis nevadensis]	-3.6259
Cluster-11327.59096	GAPDH [Locusta migratoria]	-1.1931
Cluster-11327.66942	Carnitine O-palmitoyltransferase 2, mitochondrial [Zootermopsis nevadensis]	-1.7904
Cluster-11327.56459	vitellogenin [Cyrtorhinus lividipennis]	-5.6797
Cluster-11327.87505	Larval cuticle protein 2 [Zootermopsis nevadensis]	-1.56
Cluster-11327.73499	RecName: Full=Cuticle protein 19.8; AltName: Full=LmNCP19.8 [Locusta migratoria]	-5.1062
Cluster-11327.80081	chitin synthase 1 variant B [Locusta migratoria manilensis]	-5.4903
Cluster-9853.1	Larval cuticle protein A3A [Zootermopsis nevadensis]	-3.1289
Cluster-11327.93652	Cuticle protein 7 [Zootermopsis nevadensis]	-4.8046
Cluster-11327.87865	cuticle protein 34, partial [Anopheles gambiae]	-6.8259

Cluster-11327.91780	larvae cuticle protein [Choristoneura fumiferana]	-3.7162
Cluster-11327.91785	larvae cuticle protein [Choristoneura fumiferana]	-6.5567
Cluster-11327.45053	Cuticle protein 6 [Zootermopsis nevadensis]	-2.1595

Table S7 GO enrichment analysis (Corrected *P*-value < 0.05) of the differentially expressed genes of *O. asiaticus* fed on rutin-treated food compared to those fed on no rutin-treated food.

GO terms (Corrected *P*-value < 0.05)	Up-/Down-regulation	Gene number
regulation of signal transduction	Up	17
response to oxidative stress	Up	21
defense response	Up	17
cellular response to chemical stimulus	Up	20
response to toxic substance	Up	14
regulation of immune system process	Up	13
xenobiotic metabolic process	Up	20
antioxidant activity	Up	16
oxidation-reduction process	Down	19
starch metabolic process	Down	13
sucrose metabolic process	Down	12
DNA-dependent DNA replication	Down	16
structural constituent of cuticle	Down	21
digestion	Down	26
growth factor binding	Down	14

Table S8 KEGG enrichment analysis (Corrected *P*-value < 0.05) of the differentially expressed genes in *O. asiaticus* fed on rutin-treated food compared to those fed on no rutin-treated food.

KEGG Pathway **(qValue < 0.05)**	Up-/Down- regulation	Gene number
N-Glycan biosynthesis	Down	13
Nitrogen metabolism	Down	19
Carbohydrate digestion and absorption	Down	17
Oxidative phosphorylation	Down	20
Fat digestion and absorption	Down	12
Insulin signaling pathway	Down	19
Protein digestion and absorption	Down	13
HIF-1 signaling pathway	Up	9
Metabolism of xenobiotics by cytochrome P450	Up	16
Jak−STAT signaling pathway	Up	17
FoxO signaling pathway	Up	11
AMPK signaling pathway	Up	13
Peroxisome	Up	10
Apoptosis	UP	9

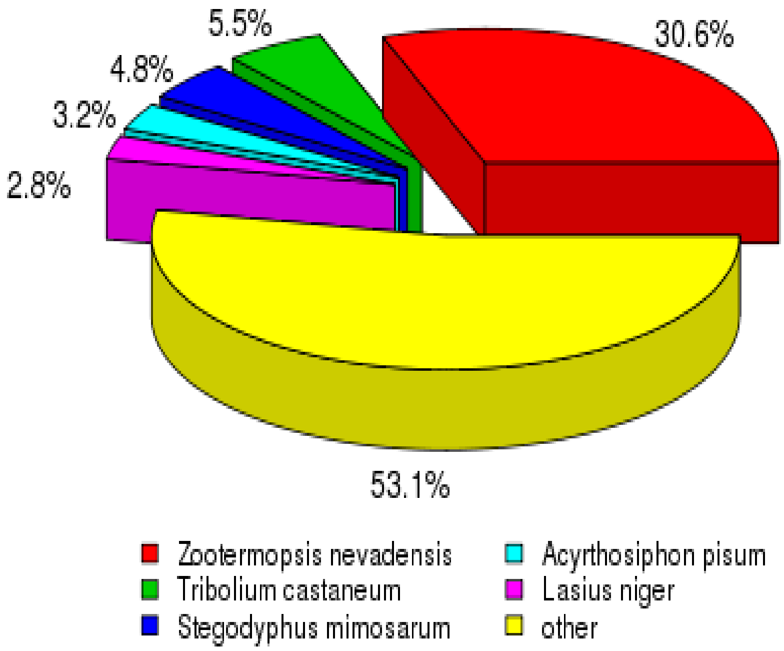

Figure S1 Species classification of Nr annotation for *O. asiaticus* sequences.
